# Acid-triggered interlayer sliding of two-dimensional copper(i)–organic frameworks: more metal sites for catalysis†

**DOI:** 10.1039/d1sc00924a

**Published:** 2021-03-19

**Authors:** Hou-Gan Zhou, Ri-Qin Xia, Ji Zheng, Daqiang Yuan, Guo-Hong Ning, Dan Li

**Affiliations:** College of Chemistry and Materials Science, Guangdong Provincial Key Laboratory of Functional Supramolecular Co-ordination Materials and Applications, Jinan University Guangzhou 510632 People's Republic of China guohongning@jnu.edu.cn danli@jnu.edu.cn; State Key Laboratory of Structure Chemistry, Fujian Institute of Research on the Structure of Matter, Chinese Academy of Sciences (CAS) Fuzhou 350002 People's Republic of China

## Abstract

The interlay sliding of two-dimensional (2D) metal–organic and covalent–organic frameworks (MOFs and COFs) affects not only the layout features of the structures, but also the functional output of the materials. However, the control of interlay stacking is the major hurdle that needs to be overcome to construct new functional layer materials. Herein, we report the preparation of a pair of isostructural 2D copper(i) organic frameworks with an eclipsed AA stacking structure, namely **JNM-3-AA**, and a staggered ABC stacking topology, denoted **JNM-3-ABC**, by combining the chemistry of MOFs and COFs. The variation of interlayer stacking largely influences their functionality, including porosity (BET surface areas of 695.61 and 34.22 m^2^ g^−1^ for **JNM-3-AA** and **JNM-3-ABC**, respectively), chemical stability, and catalytic activities (less than 10% or ∼86% yield using **JNM-3-AA** or **JNM-3-ABC** as catalysts for click reaction, respectively). More interestingly, the structure transformation from **JNM-3-ABC** to **JNM-3-AA** is readily achieved by simple addition of trifluoroacetic acid accompanied by the extension of porosities from BET surface areas of 34.22 to 441.22 m^2^ g^−1^, resulting in *in situ* acceleration of the adoption rate (removal efficiency increases from ∼10 to 99.9%), which is rarely observed in 2D MOFs and COFs.

## Abstract 2

The addition of TFA can trigger the interlay sliding of 2D copper(i) organic frameworks prepared by combing the chemistry of MOFs and COFs. The variation of interlay stacking largely affected the porosity, chemical stability and catalytic activities.

## Graphical abstract

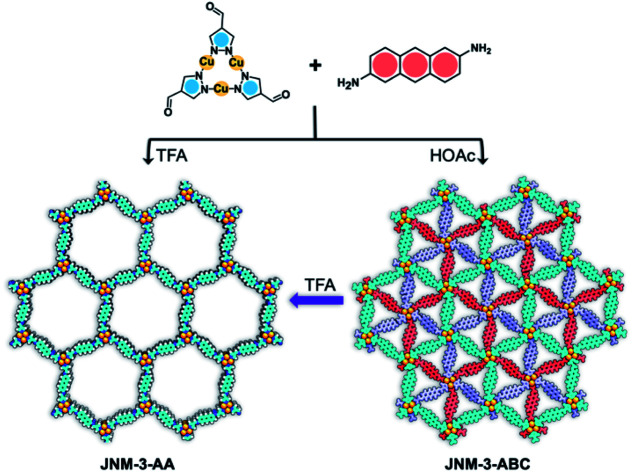

## Introduction

Two-dimensional (2D) metal–organic frameworks (MOFs) and covalent–organic frameworks (COFs) are emerging classes of layered crystalline materials. They have attracted extensive attention because of their atomically precise, periodic and diverse structures, intrinsic porosity, tunable electronic properties, and great applications in gas adsorption and separation, catalysis, sensing, electronics and energy storage.^1–11^ The three-dimensional (3D) structures of these framework materials may be constructed from 2D monolayers or sheets, stacking with each other by non-covalent interactions (*e.g.*, π–π and van der Waals interactions) to form porous channels orthogonal to the planes. The alteration of interlayer interactions and stacking can greatly affect not only structural features, such as crystallinity and porosity,^12^ but also the electronic and optical properties, including interlayer electron or charge transport, and excitation states,^13^ which has been extensively demonstrated by graphene^14,15^ and transition metal dichalcogenides.^16,17^ In contrast, few examples and strategies have been explored to control the interlayer interactions and realize interlayer sliding of 2D COFs, such as incorporation of complementary donor–acceptor interactions, solvation and desolvation, and the introduction of steric hinderance on the organic linkers.^18–23^ The interlayer stacking transformation of 2D MOFs has also rarely been achieved.^8,24,25^

**Scheme 1:**
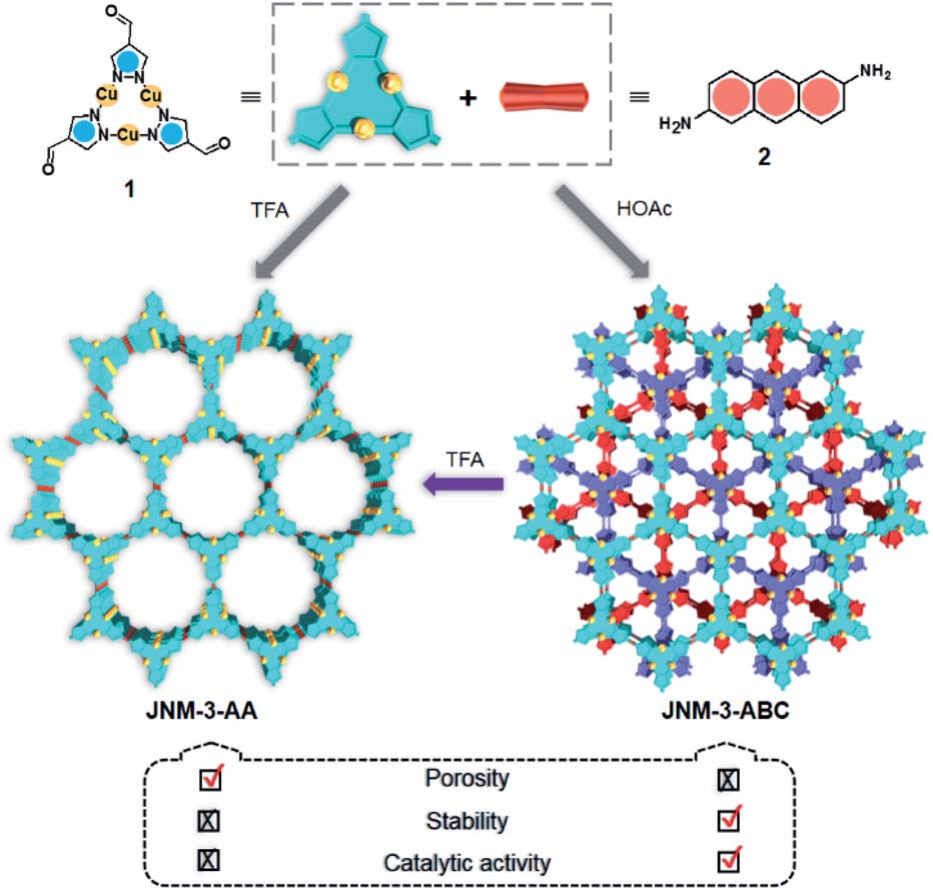
Syntheses and structure transformation illustration for **JNM-3**.

Recently, we prepared copper(i) cyclic trinuclear unit (CTU)-based 2D covalent metal–organic frameworks (CMOFs) by combining the chemistry of MOFs and COFs.^26^ The CTU-based 2D CMOFs have inherited advantages from both MOFs and COFs, including good crystallinity and stability, high porosity, and excellent catalytic activities resulting from inborn metal open sites.^26,27^ In addition, d^10^ metal CTUs can provide additional non-covalent interactions, such as metallophilic attraction,^28,29^ compared to pure organic systems, and bring merits for fine-tuning the interlayer stacking. Moreover, Cu(i) CTUs, acting as secondary building units to construct frameworks, have unsaturated metals as potential catalytic centers. Such unique structural features provide a promising platform for studying how interlayer stacking impacts the catalytic activities, which have so far not been illustrated for 2D MOFs and COFs. Herein, we demonstrate the synthetic control of the interlayer stacking structure (eclipsed AA and staggered ABC stacking), chemical stability and catalytic activities of 2D CTU-based CMOFs by acid stimulation. A 2D Cu(i) CTU-based CMOF, namely **JNM-3** (JNM represents Jinan material) was prepared from the imine condensation reaction between Cu-CTU (**1**) and anthracene-2,6-diamine (**2**) (Scheme 1). By varying the reaction conditions (*i.e.*, increasing acidity) two different stacking structures of **JNM-3**, specifically eclipsed AA stacking (denoted as **JNM-3-AA**) and staggered ABC stacking (**JNM-3-ABC**), were obtained. Interestingly, the almost non-porous **JNM-3-ABC** is able to completely transform into highly porous **JNM-3-AA** by simply adding trifluoroacetic acid (TFA). **JNM-3-ABC** exhibits higher chemical stability than **JNM-3-AA**, which is strongly related to the stacking mode. More interestingly, **JNM-3-ABC** exposes more metal-active sites than **JNM-3-AA**, resulting in much higher catalytic activities for the azide-alkyne cycloaddition (AAC) reaction. Due to the narrow pore-size distribution, the catalytic activity of **JNM-3-ABC** also declines with increase in alkyne size. Overall, combining the chemistry of MOFs and COFs allows us to fuse their advantages and explore a new strategy for precise control of interlayer stacking of 2D materials, leading to further understanding of the structure-property relationship.

## Results and discussion

### Synthesis and characterization

**JNM-3-AA** was obtained as a deep red crystalline powder through solvothermolysis of a mixture of Cu-CTU **1** and organic linker **2** in a 5 : 5 : 1 (v/v) mixture of mesitylene, 1,4-dioxane, and 6 M aqueous TFA. Replacing TFA with 6 M aqueous acetic acid (HOAc), **JNM-3-ABC** can be synthesized as a yellow crystalline powder (Scheme 1). In the Fourier-transform infrared (FT-IR) spectra of **JNM-3** (*i.e.*, **JNM-3-AA** and **JNM-3-ABC**), the disappearance of the N–H stretching signals located at 3387 to 3196 cm^−1^ and of the characteristic C=O stretching bonds (∼1667 cm^−1^), and the appearance of the C=N stretching bands located at 1633 to 1627 cm^−1^ (Fig. S3†), confirm the formation of imine linkages. The solid-state ^13^C CP/MAS NMR spectra of **JNM-3** reveal the removal of the aldehyde carbon signals of CTU **1** located at 184 ppm^26^ and the appearance of characteristic resonance peaks of imine carbons at 155 and 151 ppm, for **JNM-3-AA** and **JNM-3-ABC**, respectively, also evidencing the formation of imine bonds (Fig. S4†). In addition, scanning electron microscopy of **JNM-3-AA** and **JNM-3-ABC** displayed a layered structure and nanotube morphology, respectively (Fig. S5†). Energy dispersive X-ray spectroscopy elemental mapping of **JNM-3** illustrated the uniform distribution of C, N, and Cu atoms (Fig. S6 and S7†).

### Crystal structure

**Fig. 1:**
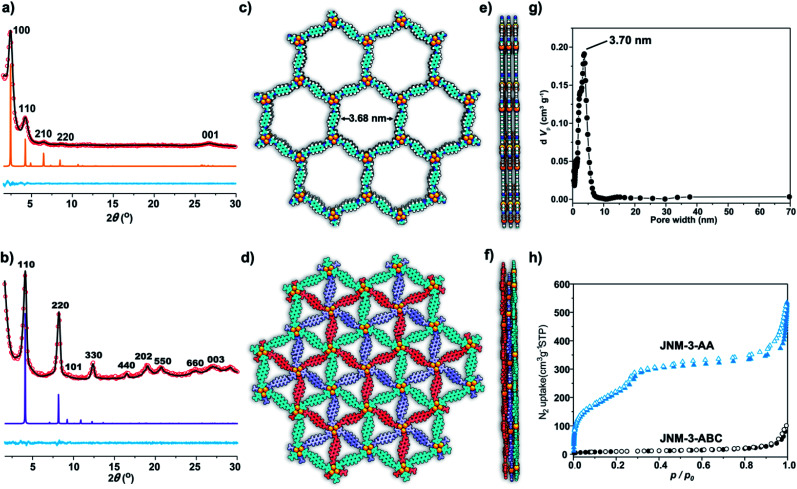
PXRD patterns of (a) **JNM-3-AA** and (b) **JNM-3-ABC** with the experimental profiles in black, difference curve in light blue, and calculated profiles of AA (orange) and ABC (purple) packing modes. (Herein, the preferred orientation along with the (110) plane was considered in the calculated ABC model, see ESI† for details). Top (c) and side (e) views of the corresponding refined 2D crystal structure of **JNM-3-AA**. Top (d) and side (f) views of the corresponding refined 2D crystal structure of **JNM-3-ABC**. (g) The pore-size distribution profiles of **JNM-3-AA** calculated by nonlocal DFT modeling based on N_2_ adsorption data, showing a uniform pore size of ∼3.70 nm. (h) The N_2_ adsorption (filled) and desorption (open) isotherm profiles of **JNM-3-AA** and **JNM-3-ABC** at 77 K.

The crystal structure of **JNM-3** was determined by the powder X-ray diffraction (PXRD) experiments and theoretical simulations. As shown in Fig. 1a, the PXRD patterns of **JNM-3-AA** show a major peak at 2.43°, which corresponds to the (100) reflection plane, accompanied by four minor peaks at 4.22, 6.47, 8.46, and 26.85° attributed to (110), (210), (220), and (001) diffractions, respectively. In sharp contrast, for **JNM-3-ABC**, two intense peaks corresponding to the (110) and (220) reflection planes appear at ∼4.1 and 8.2°, along with several minor peaks at 9.5, 12.3, 16.3, 18.9, 20.4, 24.6, and 26.9° attributed to (101), (330), (440), (202), (550), (660), and (003) diffractions, respectively (Fig. 1b). To elucidate the structure of **JNM-3**, three possible 2D structures with different stacking models, that is, eclipsed stacking (AA) and staggered stacking (AB and ABC) models (Fig. S10–S12†), have been simulated using Material Studio (see ESI for details†). In addition, the experimental PXRD patterns of **JNM-3-AA** agree well with the simulated AA stacking model in the *P*6 space group (Fig. 1a and S8†). However, the stacking model proposed for the PXRD patterns of **JNM-3-ABC** matched well with the calculated patterns of the ABC stacking model in the *R-*3 space group, considering the preferred orientation along the (110) direction (Fig. 1b and S9, see ESI for details†). Furthermore, Pawley refinements give optimized unit cell parameters (*a* = *b* = 41.91 Å, *c* = 4.22 Å for **JNM-3-AA** and *a* = *b* = 43.36 Å, *c* = 9.93 Å for **JNM-3-ABC**) and good agreement parameters (*R*_p_ = 2.78% and *R*_wp_ = 3.44% for **JNM-3-AA** and *R*_p_ = 3.59% and *R*_wp_ = 4.88% for **JNM-3-ABC**). The refined PXRD patterns match well with the experimental PXRD data, as confirmed by the negligible difference plot in Fig. 1a and b.

The porosity of **JNM-3** has been examined by nitrogen adsorption measurements at 77 K (Fig. 1h). **JNM-3-AA** illustrates type IV adsorption curves featuring a mesoporous nature and the Brunauer–Emmett–Teller (BET) surface areas are calculated to be 695.61 m^2^ g^−1^. In contrast, **JNM-3-ABC** exhibits type I adsorption curves featuring a microporous nature and the BET surface areas are as low as 34.22 m^2^ g^−1^. Nonlocal density functional theory (NLDFT) suggests a narrow pore-size distribution with an average pore width of ∼3.71 and ∼1.5 nm for **JNM-3-AA** and **JNM-3-ABC**, respectively, which is in good agreement with the simulated values from the eclipsed AA (∼3.68 nm) and staggered ABC model (∼1.3 nm), respectively (Fig. 1g and S10†). The pore and stacking structures of **JNM-3** were also examined by transmission electron microscopy (TEM) (Fig. S16†). Interestingly, the TEM images of **JNM-3-AA** reveal a clear hexagonal structure with a diameter of ∼3.50 nm, further supporting the eclipsed AA stacking model (Fig. S15a and b†). In addition, the TEM images of **JNM-3-ABC** display highly ordered, straight black-and-white stripes (Fig. S15c and d†) with a space distance of ∼2.2 nm attributed to the projections of the channels along the (110) reflection plane, evidencing the staggered ABC stacking structure.

### Chemical stabilities

The alteration of the interlayer stacking indeed influences the chemical stability. **JNM-3-ABC** exhibits higher stability towards heat, light, water, and base than **JNM-3-AA**. Thermogravimetric analysis (TGA) and variable-temperature (VT)-PXRD reveal that **JNM-3-ABC** has better thermal stabilities and remains crystalline up to 320 °C, while **JNM-3-AA** decomposes at ∼250 °C (Fig. S16–S19†). In addition, the crystallinity of **JNM-3-AA** gradually decreases upon exposure to light, which is probably caused by the dimerization of anthracene units under light irradiation as reported previously.^30^ However, due to the displacement of anthracene units in the staggered ABC stacking mode (Fig. 1d), **JNM-3-ABC** is insensitive towards light and maintains its crystallinity. Moreover, in spite of the common observation of fast oxidation and decomposition of Cu(i) CTU-based compounds when exposed to air,^31^ both **JNM-3-AA** and **JNM-3-ABC** exhibit superior stability towards air over one month, and the Cu(i) ions remain intact as evidenced by the X-ray photoelectron spectroscopy measurements (Fig. S20 and S21†). Furthermore, **JNM-3-ABC** features better solvent and base stabilities than **JNM-3-AA**. For instance, the crystallinity of **JNM-3-ABC** is retained upon suspension in various organic solvents, water, and even NaOH solutions for 3 days, as confirmed by PXRD analyses, while the crystallinity of **JNM-3-AA** decreases in polar organic solvents, including acetonitrile, dioxane, and DMF, and completely vanishes in water and NaOH solutions (Fig. S22 and S23†).

### Structure transformation

**Fig. 2:**
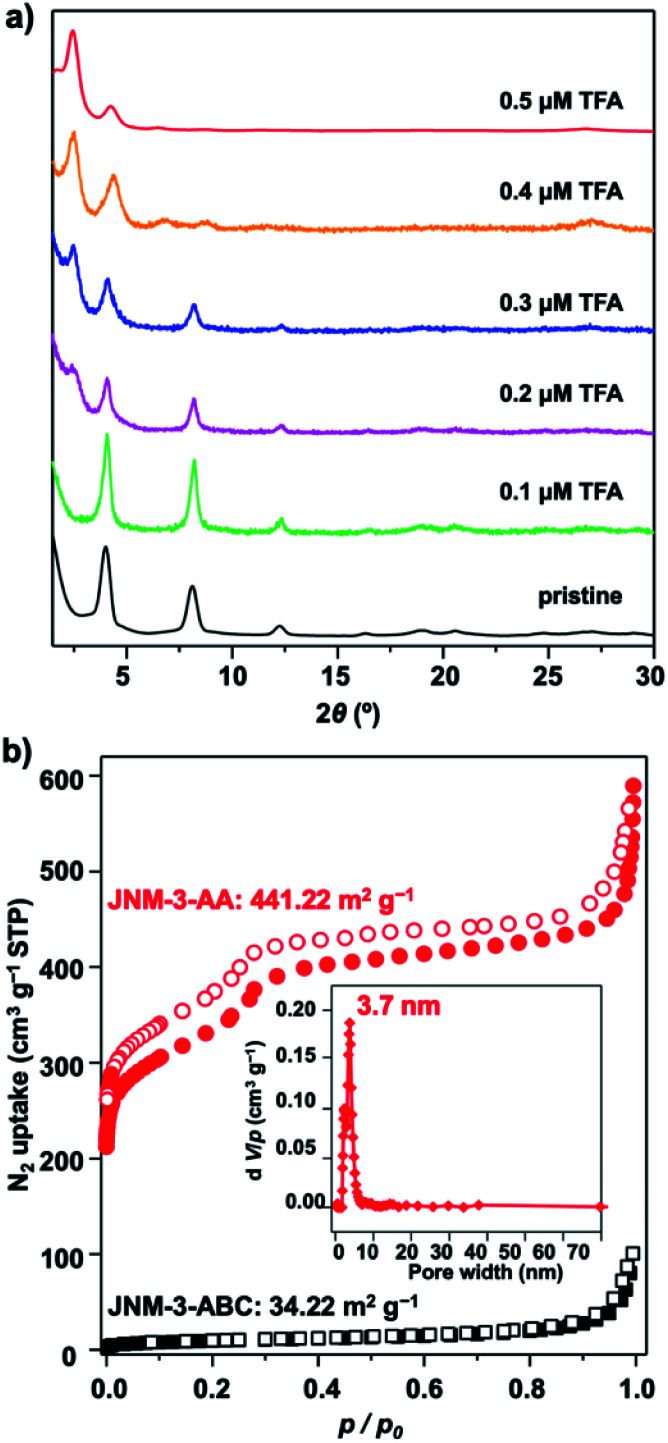
(a) Monitoring the interlayer stacking transformation by addition of varied concentration of TFA (black, pristine; green to red, 0.1 to 0.5 μM TFA). (b) The N_2_ adsorption (filled) and desorption (open) isotherm profiles of **JNM-3-AA** (red cycle) transferred from **JNM-3-ABC** by addition of 0.5 μM TFA and refresh-prepared **JNM-3-ABC** (black square) at 77 K (inset, the pore-size distribution profiles of **JNM-3-AA** showing a uniform pore size of ∼3.7 nm.).

To further understand the role of TFA in control of the interlayer stacking, the structure transformation processes were monitored by tuning the concentration of TFA. Generally, **JNM-3-ABC** was soaked in a mixture solution of mesitylene, 1,4-dioxane, and aqueous TFA for 0.5 hour at room temperature (rt) and then the PXRD of the resulting powders were recorded. As shown in Fig. 2a, the peak at 2.43° corresponding to the characteristic signal of **JNM-3-AA** appeared and the peaks at ∼4.1, 8.2, 9.5, 12.3, 16.3, 18.9, and 20.4° assigned to characteristic signal of **JNM-3-ABC** decreased and then vanished upon increasing the TFA concentration from 0 to 0.5 μM, resulting in the structure transformation from a staggered ABC to an eclipsed AA stacking model. More importantly, the nitrogen adsorption and desorption profiles, BET surface areas (441.22 m^2^ g^−1^), and the pore-size distribution (∼3.7 nm) of the transformed **JNM-3-AA** are similar to those for freshly prepared **JNM-3-AA**, further supporting the complete transformation of the interlayer stacking (Fig. 2b). To the best of our knowledge, such a great increment in porosity (∼10 times enhancement) by simple addition of acid has never been achieved in crystalline porous materials. The immersion of **JNM-3-AA** into a MeOH solution of NaOMe cannot trigger this structure transformation and only slightly reduces the crystallinity, indicating that strong Cu–Cu interactions lock the structure into the eclipsed stacking mode (Fig. S27†).

Theoretical calculations were performed to investigate the structure transformation mechanism using Material Studio with the DMol^3^ molecular dynamics module.^18,32^ On the one hand, the total crystal stacking energy of AA stacking (46.59 kcal mol^−1^) is much higher than that for ABC stacking (39.91 kcal mol^−1^), which indicates that the AA stacking mode is energetically favorable. In addition, the simulation results suggest that **JNM-3-AA** cannot transform to **JNM-3-ABC** after neutralization with base (Table S4†), which is in good agreement with the experimental results. It is known that the Schiff-base units in COFs can be protonated after adding TFA,^33^ thus the protonated model was also calculated using the same method. The total crystal stacking energy of the protonated AA stacking model (65.14 kcal mol^−1^) is also higher than that for ABC stacking (60.85 kcal mol^−1^), suggesting that the AA stacking mode is energetically favorable after addition of TFA (Table S5†). These results are in good agreement with the experimental observations and further confirm that the stacking transformation can be simply and efficiently triggered by adding acid.

**Fig. 3:**
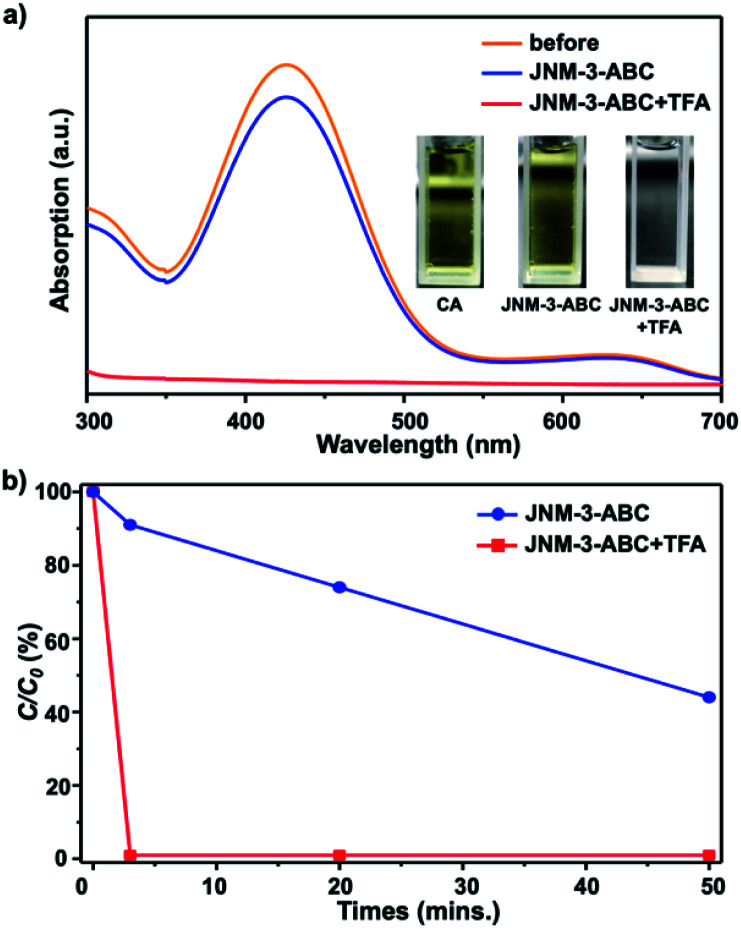
(a) UV-visible absorption spectra of an aqueous solution of CA indicating slight decrease or disappearance of absorption intensity after treatment with **JNM-3-ABC** or **JNM-3-ABC** and TFA, respectively (orange and blue line represents before and after treatment with **JNM-3-ABC** at 3 min; red line represents after addition of TFA into the mixture of **JNM-3-ABC** and CA for 3 min; inset, the photographs of CA, and filtrate after treatment of only **JNM-3-ABC** or **JNM-3-ABC** and TFA, showing yellow, yellow, and colorless). (b) Change in concentration of CA over time after treatment with **JNM-3-ABC** or **JNM-3-ABC** and TFA, determined by the change in absorbance relative to the initial absorbance (*C*/*C*_*0*_).

To verify that the structure transformation of **JNM-3** can affect the adsorption ability, adsorption experiments with an organic dye, chrome azurol S (CA), were performed. The molecular diameter of CA of ∼1.4 nm is much smaller than the pore size of **JNM-3-AA**, but close to that of **JNM-3-ABC**.^34^ The adsorption experiments of CA were conducted by immersing the **JNM-3-ABC** (10 mg) in an aqueous solution of CA (100 μM, 3 mL) without or with TFA (6 M, 0.2 mL), and the concentration of CA was monitored by UV-visible spectroscopy. As expected, **JNM-3-ABC** slowly adsorbs CA and exhibits a slow adsorption rate (∼50 min) with 50% removal efficiency (Fig. 3b). However, when TFA was added into the mixture of CA and **JNM-3-ABC**, a much higher adsorption rate (∼3 min) with 99.9% removal efficiency was observed (Fig. 3), due to the *in situ* extension of the pore size caused by the structure transformation from **JNM-3-ABC** to **JNM-3-AA**.

### Heterogeneous catalysis

It is proposed that catalytic activities of COFs and MOFs are highly related to the density of metal open sites or unsaturated metal centers, and the porosity.^35–38^ Since the theoretical ratio of copper(i) density between **JNM-3-ABC** and **JNM-3-AA** is 1.2 (see ESI for details), and **JNM-3-AA** has a much higher porosity than **JNM-3-ABC**, the isostructural pair of 2D **JNM-3**s provide a promising platform to investigate the relationship between the density of metal open sites as well as the porosity and catalytic activities of 2D MOFs. Therefore, the azide-alkyne cycloaddition (AAC) reaction catalyzed by **JNM-3**s was studied *via* an initial exploration of a model reaction of methyl 2-azidoacetate (**3**) and phenyl acetylene (**4**). As shown in Tables 1 and S6,† at rt, and in dichloromethane (DCM), the mixture of **3**, **4** and **JNM-3-ABC** (4 mol%, based on Cu-CTU) efficiently formed the cycloaddition product in 12 h, with ∼81% conversion, while **JNM-3-AA** only gives ∼10% conversion. These observations suggested that more with metal open sites, higher catalytic activities can be achieved at rt. However, when the reaction mixture is heated to 60 °C, **JNM-3-AA** gives a conversion of 90%. This is because the harsh conditions might promote exfoliation or increase the defects of the 2D materials, thus exposing more metal open sites, and leading to enhancement of the catalytic activities.

**Table 1:** **JNM-3** catalyzed azide-alkyne cycloaddition reaction^a^

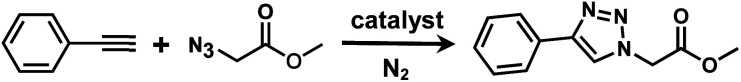				
Entry	Catalyst	Solvent	Temp. (°C)	Conversion^b^ (%)
1	**JNM-3-AA**	CH_2_Cl_2_	rt	10
2	**JNM-3-ABC**	CH_2_Cl_2_	rt	81
3	**JNM-3-AA**	*n*-BuOH	60	90
4	**JNM-3-ABC**	CH_2_Cl_2_	40	93
5	CTU **1**	CH_2_Cl_2_	rt	99
6	None	CH_2_Cl_2_	rt	0

a Reaction conditions: methyl 2-azidoacetate (0.6 mmol), phenyl acetylene (0.5 mmol), catalysts loading base on Cu-CTU (4 mol%); solvent (3 mL) and 12 h.

b The reported conversions are based on GC-MS analysis.

Interestingly, increasing the molecular size of the alkyne substrates significantly reduced the reaction yield with **JNM-3-ABC**, while **JNM-3-AA** still showed similar yields. For instance, by changing the alkyne substrates from **4** (Table 2, entry 1) to 2-ethynylnaphthalene (Table 2, entry 4), the yield of cycloaddition product catalyzed with **JNM-3-ABC** sharply decreased from 79% to 36%, but, as shown in Tables 2 and S7,† for **JNM-3-AA** no obvious change has been observed (*i.e.*, ∼10% and ∼90% at rt and 60 °C, respectively). More interestingly, the reverse reactivities have been found when the substrates are changed from aryl acetylene to alkyl acetylene. In particular, **JNM-3-ABC** exhibits much higher catalytic performance for the AAC reaction using alkyl acetylene than for those of CTU **1** (Tables 2 and S7,† entries 5–7), while CTU **1** shows higher activities for aryl acetylene than those of **JNM-3-ABC** (Tables 2 and S7,† entries 1–4). For examples, for cyclopropyl acetylene, the isolated yields of the cycloaddition product are 86% and 40% for **JNM-3-ABC** (Table 2, entry 5) and CTU **1** (Table S7,† entry 5), respectively. These results revealed that the density of metal open sites is the dominant factor for catalytic efficiency compared to the porosity at rt. Meanwhile, the porosity can also affect the catalytic activities, especially when the size of the substrates is close to or larger than the pore size of the catalysts.

**Table 2:** Effects of substrate size on the **JNM-3** catalyzed AAC reactions^a^

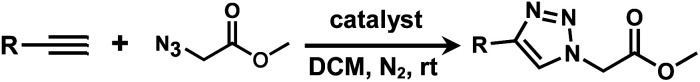			
Entry	Substrate	Product yield (%)	
		**JNM-3-ABC** b	**JNM-3-AA** c
1	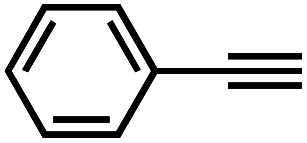	79	8
2	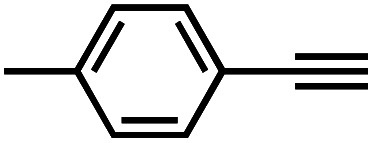	70	11
3	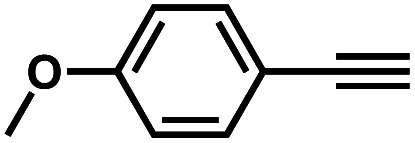	66	10
4	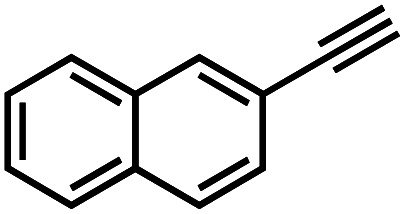	36	13
5	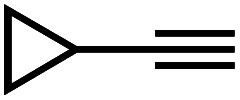	86	9
6	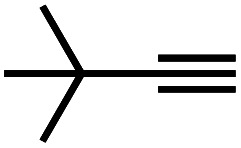	65	4
7	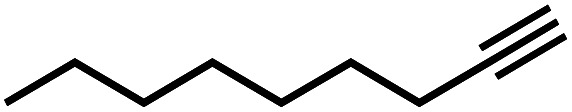	10	2

a Reaction conditions: methyl 2-azidoacetate (0.6 mmol), phenyl acetylene (0.5 mmol), catalysts loading base on Cu-CTU (4 mol%); solvent (3 mL) and N_2_ atm., 12 h.

b The reported yields here are based on isolated yield.

c The reported yields here are based on GC-MS analysis, respectively.

## Conclusions

In summary, we have synthesized a 2D copper(i) organic framework, namely **JNM-3**, by combining the chemistry of MOFs and COFs. The interlayer stacking of **JNM-3** can be tuned by variation of the reaction conditions, and the eclipsed AA stacking structure, **JNM-3-AA**, as well as the staggered ABC stacking architecture, **JNM-3-ABC**, are obtained. The interlayer stacking structure of **JNM-3** largely impacts its functionality, including porosity, chemical stability and catalytic activities. For instance, **JNM-3-AA** exhibits larger porosity, with BET surface areas of 695.61 m^2^ g^−1^, lower chemical stability towards water and base, and lower catalytic activities for AAC reaction, while the **JNM-3-ABC** shows lower porosity (34.22 m^2^ g^−1^), good stability, and superior catalytic activities. More interestingly, the structure transformation from **JNM-3-ABC** to **JNM-3-AA** is readily achieved by simple addition of TFA accompanied by the extension of the porosities from BET surface areas of 34.22 to 441.22 m^2^ g^−1^, which is rarely observed in 2D MOFs and COFs. Our findings reveal the influence of interlayer stacking on the functionality of 2D materials and might pave the way for designing smart 2D materials with desirable properties.

## Author contributions

G.-H. N., and D. L. designed the research; H.-G. Z. conducted the experiments and data analysis; R.-Q. X. contributed to catalytic studies; J. Z., and D. Q. contributed to structural refinement; H.-G. Z., G.-H. N., and D. L. co-wrote the manuscript. All authors read and commented on the manuscript.

## Conflicts of interest

There are no conflicts to declare.

## Supplementary Material

SC-012-D1SC00924A-s001
