# Supplementary material for: Acid-triggered interlayer sliding of two-dimensional copper(i)–organic frameworks: more metal sites for catalysis
Source: Chem Sci. 2021 Mar 19;12(18):6280–6. doi: 10.1039/d1sc00924a (PMC8115315; doi:10.1039/d1sc00924a)
Supplement: SC-012-D1SC00924A-s001 [file SC-012-D1SC00924A-s001.pdf]

Electronic Supplementary Material (ESI) for Chemical Science.
This journal is © The Royal Society of Chemistry 2021

Electronic Supplementary Material (ESI) for Chemical Science.
This journal is © The Royal Society of Chemistry 2021

Electronic Supplementary Information (ESI) for

Acid-Triggered Interlayer Sliding of Two-Dimensional Copper(I)-Organic Frameworks: More Metal Sites for Catalysis

Hou-Gan Zhou,[a] Ri-Qin Xia,[a] Ji Zheng,[a] Daqiang Yuan,[b] Guo-Hong Ning*[a] and Dan Li*[a]

[a]College of Chemistry and Materials Science, and Guangdong Provincial Key Laboratory of Functional Supramolecular Coordination Materials and Applications, Jinan University, Guangzhou 510632, People's Republic of China

[b]State Key Laboratory of Structure Chemistry, Fujian Institute of Research on the Structure of Matter, Chinese Academy of Sciences (CAS), Fuzhou 350002, People's Republic of China

E-mail: guohongning@jnu.edu.cn; danli@jnu.edu.cn

Contents

1. Instruments and methods.

2. Synthetic procedures

3. Fourier-transform infrared (FT-IR) spectra

4. Solid-state ^{13}C CP/MAS NMR spectra

5. Scanning electron microscopy (SEM)

6. Energy Dispersive X-ray Spectroscopy (EDS)

7. Structural Simulation

8. Gas adsorption isotherms and the pore size distribution

9. Transmission electron microscopy (TEM)

10. Thermogravimetric analysis (TGA)

11. Various-Temperature PXRD

12. X-ray photoelectron spectroscopy (XPS)

13. Stability in various solvents

14. Structure transformation

15. Dye adsorption experiments

16. Catalysis for Click reaction

1. Instruments and methods.

Starting materials, reagents, and solvents were purchased from commercial sources and used without further purification. Powder X-ray diffraction (PXRD) data was collected at 40 kV, 30 mA using microcrystalline samples on a Rigaku Ultima IV diffractometer using Cu-Kα radiation (λ = 1.5418 Å). The measurement parameters include a scan speed of 0.5 °/min, a step size of 0.02°, and a scan range of 2θ from 1.5°to 30°. For temperature-dependent PXRD, the measurement parameters include a scan speed of 2 °C/min, a step size of 0.02°, and a scan range of 2θ from 1.5° to 30°. Thermogravimetric analysis was performed on a Mettler-Toledo (TGA/DSC1) thermal analyzer. Measurement were made on approximately 5 mg of dried samples under a N_2 flow with a heating rate of 10 °C/min. The scanning electron microscopy (SEM) and Energy Dispersive X-ray Spectroscopy (EDS) images were obtained on a Helios Nanolab G3 CX microscope. Transmission electron microscopy (TEM) analysis was performed on a JEM-2100F. Fourier transform infrared (FT-IR) spectrum was measured using a Nicolet Avatar 360 FT-IR spectrophotometer. X-ray photoelectron spectroscopy (XPS) experiments were performed by a Thermo ESCALAB 250XI system. GC-MS analysis was carried out on an Agilent 7890B GC analyzer. Liquid ^{1}H and ^{13}C NMR spectra were recorded on a Bruker Biospin Avance (400 MHz) equipment using tetramethylsilane (TMS) as an internal standard. Solid-state NMR experiments were performed on a Bruker WB Avance II 400 MHz NMR spectrometer. The ^{13}C CP/MAS NMR spectra were recorded with a 4-mm double-resonance MAS probe and with a sample spinning rate of 10.0 kHz; a contact time of 2 ms (ramp 100) and a pulse delay of 3 s was applied. Gas sorption analyses were conducted using an ASAP 2020 PLUS Analyzer (Micromeritics) with extra-high pure gases. Surface areas were calculated from the adsorption data using Brunauer-Emmett-Teller (BET) methods. The pore size distribution curves were obtained from the adsorption branches using density functional theory (DFT) method.

2. Synthetic procedures

2-1. Synthesis of JNM-3-AA

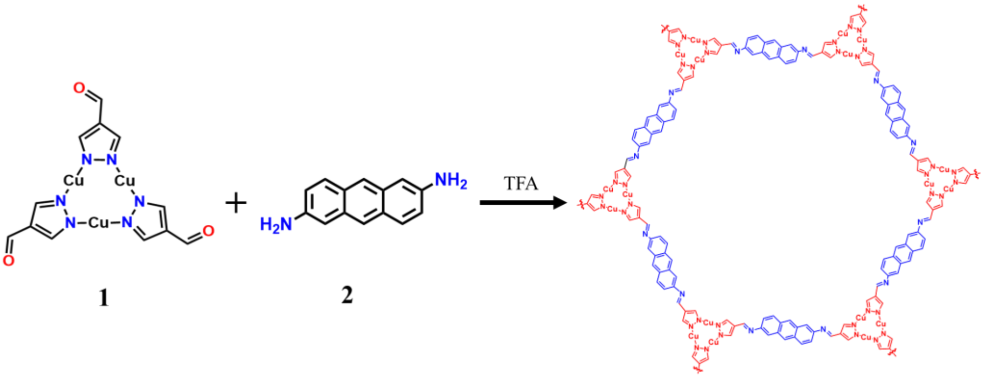

1 2

JNM-3-AA

A 10 mL Schlenk tube was charged with **1** (23.7 mg, 0.05 mmol), **2** (16.6 mg, 0.08 mmol), 0.5 mL of mesitylene, 0.5 mL of 1,4-dioxane and 0.1 mL of 6 M aqueous trifluoroacetic acid (TFA). The mixture was flash frozen at 77 K in liquid nitrogen bath and degassed with three freeze-pump-thaw cycles. Upon warming to room temperature, the mixture was heated at 120 °C for 72 h. The red powders were isolated by filtration, washed and solvent exchanged with EtOH, DMF and acetone. The resultants were dried under vacuum at 100 °C for 8 h. Yield: 34.5 mg (85.6%, based on **1**). Elemental analysis calcd (%): C 53.8, H 3.2, N 17.1; Found: C 46.3, H 3.1, N 10.9.

2-2. Synthesis of JNM-3-ABC

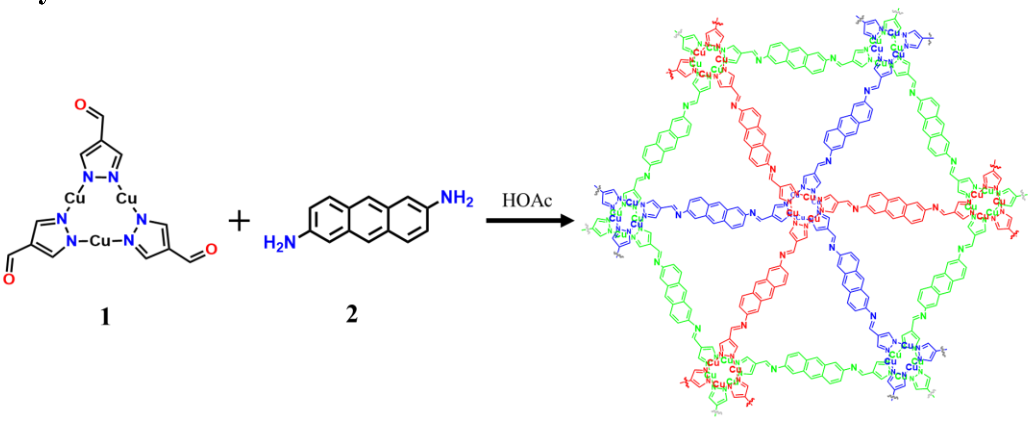

1 2

JNM-3-ABC

A 10 mL Schlenk tube was charged with **1** (23.7 mg, 0.05 mmol), **2** (16.6 mg, 0.08 mmol), 0.75 mL of *o*-dichlorobenzene (*o*-DCB), 0.25 mL of *n*-BuOH and 0.1 mL of 6 M aqueous acetic acid (HOAc). The mixture was flash frozen at 77 K in liquid nitrogen bath and degassed with three freeze-pump-thaw cycles. Upon warming to room

temperature, the mixture was heated at 100 °C for 72 h. The yellow solid was isolated by filtration, washed and solvent exchanged with EtOH, DMF and acetone. The resultants were dried under vacuum at 100 °C for 8 h. Yield: 31.1 mg (77.1%, based on 1). Elemental analysis calcd (%): C 53.8, H 3.2, N 17.1; Found: C 50.7, H 3.2, N 15.2.

2-3. Optimization of synthetic condition

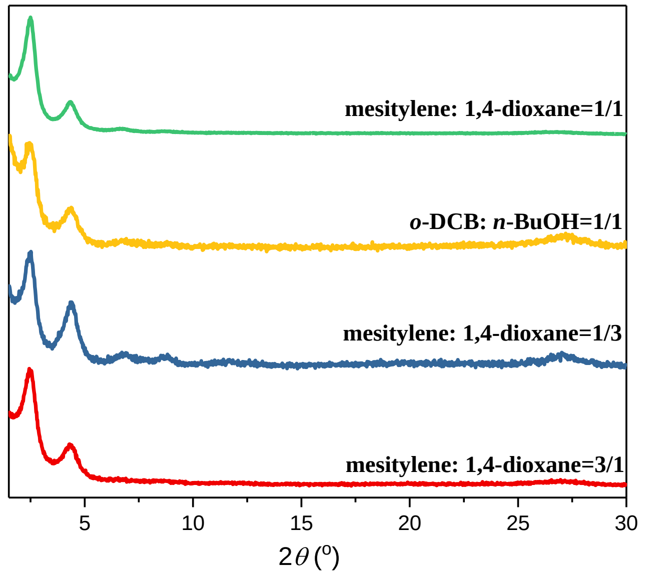

Figure S1. Optimization of **JNM-3-AA** reaction condition, showing the best condition is mesitylene (0.5 mL), 1,4-dioxane (0.5 mL) and TFA (6 M, 0.1 mL) for 3 days.

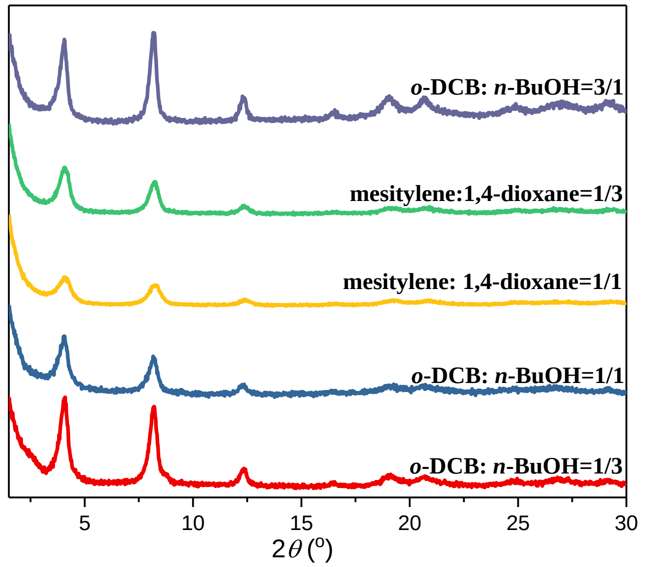

Figure S2. Optimization of **JNM-3-ABC** reaction condition, showing the best condition is *o*-dichlorobenzene (0.75 mL), *n*-BuOH (0.25 mL) and aqueous acetic acid (6 M, 0.1 mL) for 3 days.

3. Fourier-transform infrared (FT-IR) spectra

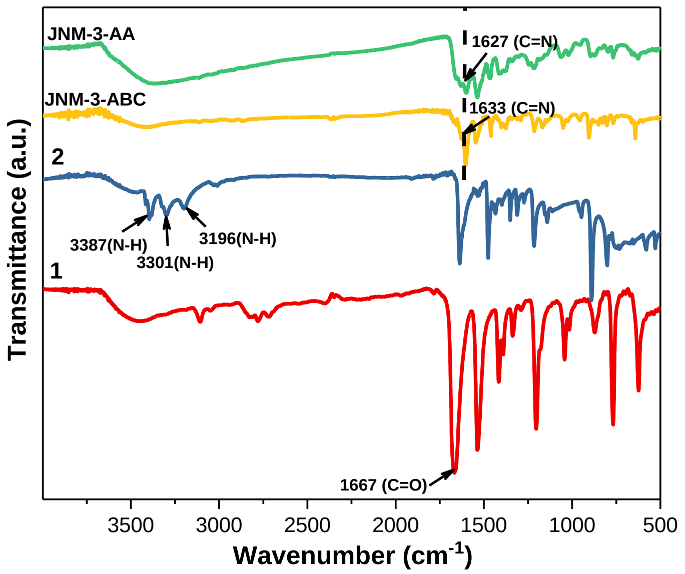

Figure S3. FT-IR spectra of **1**, **2**, **JNM-3-AA** and **JNM-3-ABC**.

4. Solid-state ^{13}C CP/MAS NMR spectra

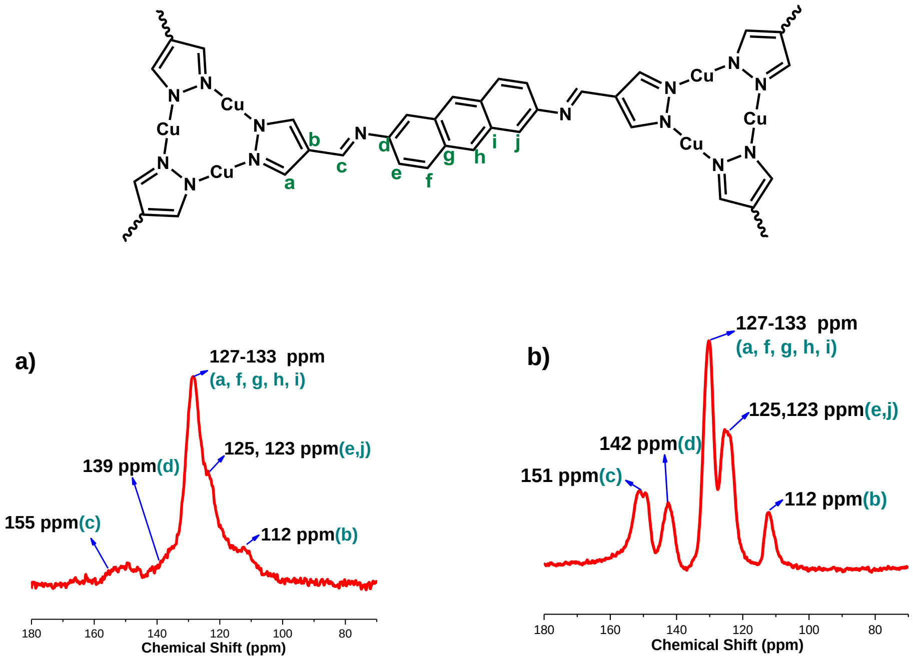

Figure S4. Solid-state ^{13}C CP/MAS NMR spectra and peak assignments of (a) **JNM-3-AA** and (b) **JNM-3-ABC**.

5. Scanning electron microscopy (SEM)

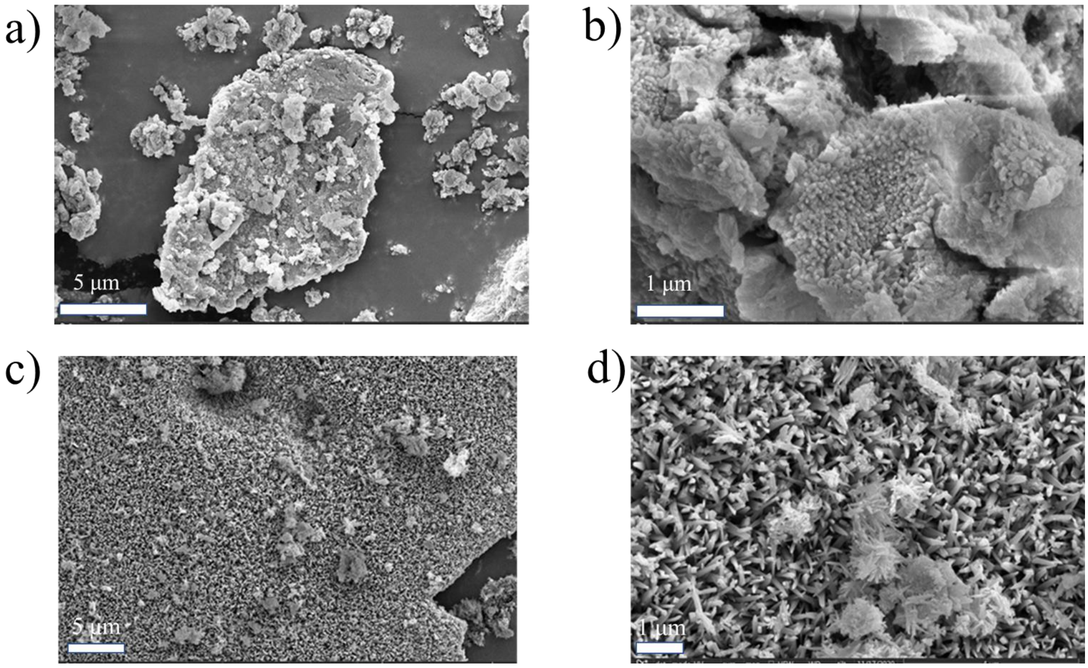

Figure S5. SEM images of (a and b) **JNM-3-AA** and (c and d) **JNM-3-ABC**.

6. Energy Dispersive X-ray Spectroscopy (EDS)

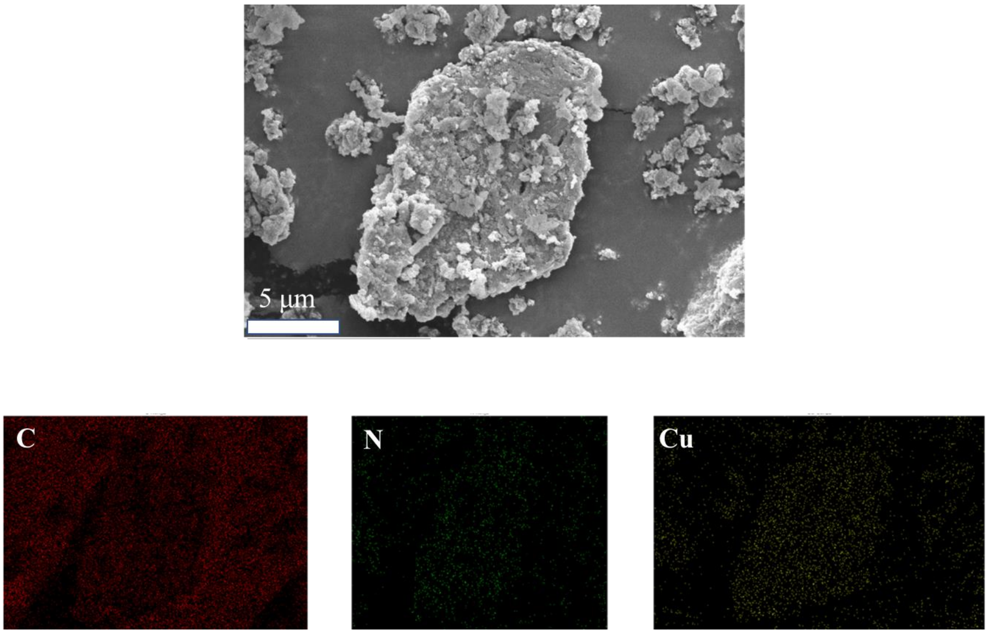

Figure S6. EDS of **JNM-3-AA** showing the uniform distribution of element C, N and Cu.

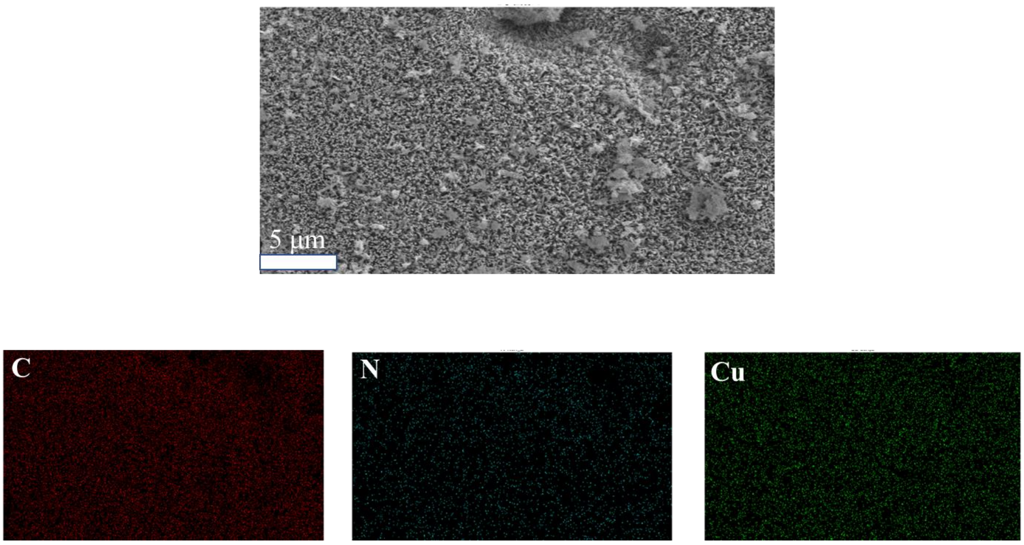

Figure S7. EDS of **JNM-3-ABC** showing the uniform distribution of element C, N and Cu.

7. Structural Simulation of JNM-3

Theoretical simulations of **JNM-3** were carried out in Accelrys Materials Studio 2019 software package. The relative total energies were calculated by molecular mechanics calculation using DMol^3 energy task, after which the simulated PXRD patterns were determined by the Reflex module. The Pawley refinement of the experimental PXRD was conducted with the Reflex module.[1-2] Considering the preferred orientation of **JNM-3-ABC,** ABC stacking models without and with the preferred orientation along (110) plane, denoted as calculated ABC and ABC-110 were simulated. The consideration of preferred orientation in simulated model only affected the relative intensities of peaks.

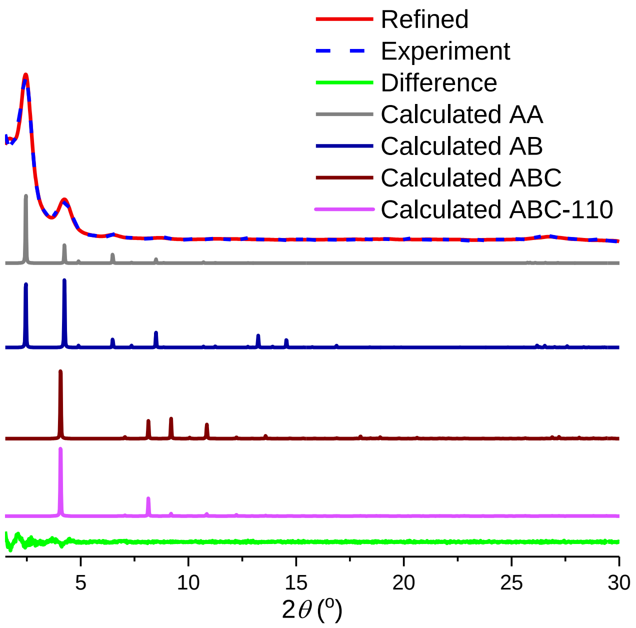

Figure S8. PXRD patterns of **JNM-3-AA** with the experimental profiles in blue, difference curve in green, and calculated profiles of AA (gray), AB (royal), ABC (wine) and ABC-110 (purple) packing modes.

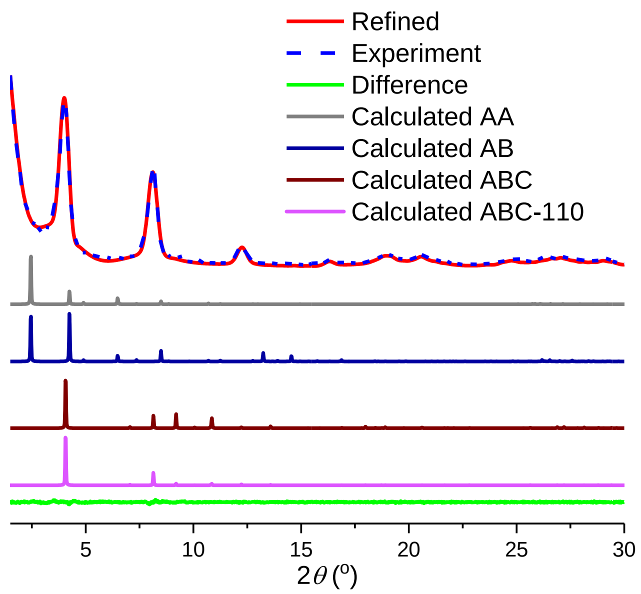

Figure S9. PXRD patterns of **JNM-3-ABC** with the experimental profiles in blue, difference curve in green, and calculated profiles of AA (gray), AB (royal), ABC (wine) and ABC-110 (purple) packing modes.

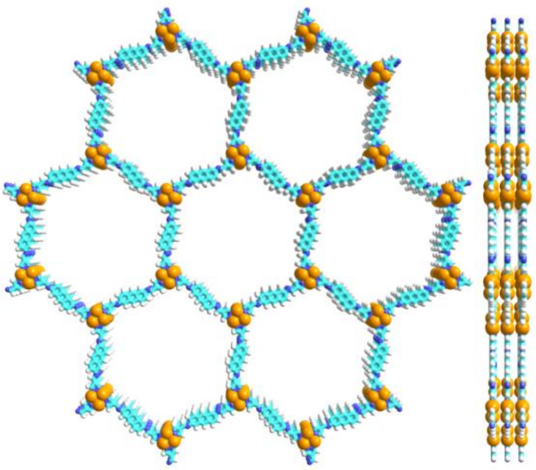

Figure S10. Space-filling mode of **JNM-3** in AA stacking model viewed from (left) *c* axis and (right) *a* axis.

Table S1. Atomic coordinates of the AA-stacking mode of **JNM-3**

Space group: *P*6<br>$a = b = 41.61$ Å, and $c = 4.18$ Å<br>$\alpha = \beta = 90°$, and $\gamma = 120°$			
	X	Y	Z
Cu1	0.61601	0.29933	1.5
C2	0.39686	-0.22298	1.5
C3	0.33864	-0.23503	1.5
C4	0.37545	-0.20539	1.5
C5	0.40837	-0.08791	1.5
C6	0.43289	-0.1029	1.5
C7	0.47094	-0.07816	1.5
C8	0.48601	-0.03911	1.5
C9	0.61279	0.16629	1.5
N10	0.33809	-0.26767	1.5
N11	1.26005	0.63454	1.5
N12	0.42184	-0.1407	1.5
H13	1.21041	0.63725	1.5
H14	0.68716	0.23442	1.5
H15	0.37838	-0.10645	1.5
H16	0.48949	-0.08965	1.5
H17	0.40299	-0.03935	1.5
H18	0.45645	0.02499	1.5
H19	0.52446	0.36415	0.5

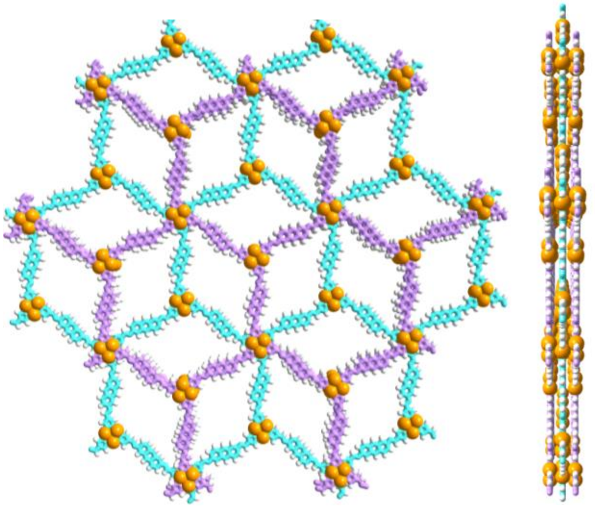

Figure S11. Space-filling mode of **JNM-3** in AB stacking model viewed from (left) *c* axis and (right) *a* axis.

Table S2. Atomic coordinates of the AB-stacking mode of **JNM-3**

Space group: $P6_3$<br>$a = b = 41.61$ Å, and $c = 6.80$ Å<br>$\alpha = \beta = 90°$, and $\gamma = 120°$			
	X	Y	Z
Cu1	0.94942	0.966	0.25
Cu2	0.71742	1.3674	0.25
C3	0.7302	1.44373	0.25
C4	0.67194	1.43166	0.25
C5	0.70877	1.46131	0.25
C6	0.74166	1.57886	0.25
C7	0.76619	1.56385	0.25
C8	0.80426	1.58857	0.25
C9	0.81935	1.62765	0.25
C10	0.79433	1.64235	0.25
C11	0.75534	1.6165	0.25
C12	0.80882	0.68084	0.25
C13	0.94632	0.83308	0.25
C14	0.93657	0.88974	0.25
C15	0.99495	0.90184	0.25
C16	0.95806	0.87217	0.25
C17	0.92506	0.75467	0.25
C18	0.90053	0.76968	0.25
C19	0.86246	0.74497	0.25
C20	0.84734	0.70589	0.25
C21	0.87236	0.69119	0.25
C22	0.91135	0.71702	0.25
C23	0.72049	1.50042	0.25
C24	0.85786	1.6527	0.25
N25	0.6714	1.39903	0.25
N26	0.59335	1.30119	0.25
N27	0.75515	1.52602	0.25
N28	0.99536	0.93437	0.25
N29	1.07323	1.03221	0.25
N30	0.91161	0.80752	0.25
H31	0.54374	1.30393	0.25
H32	1.0208	0.90131	0.25
H33	0.71167	1.56033	0.25
H34	0.82281	1.5771	0.25
H35	0.73628	1.62741	0.25
H36	0.78979	0.69178	0.25
H37	1.12266	1.02925	0.25

H38	0.64615	1.43229	0.25
H39	0.95505	0.77318	0.25
H40	0.8439	0.75645	0.25
H41	0.9304	0.70611	0.25
H42	0.8769	1.64176	0.25
H43	0.85774	1.03068	0.25
H44	0.80894	1.30256	0.25

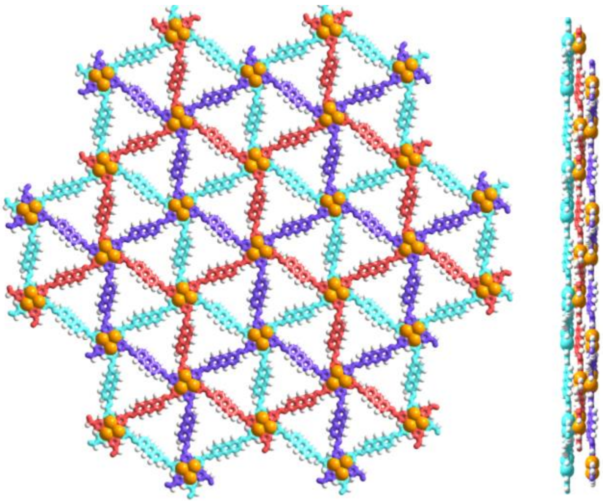

Figure S12. Space-filling mode of **JNM-3** in ABC stacking model viewed from (left) *c* axis and (right) *a* axis.

Table S3. Atomic coordinates of the ABC-stacking mode of **JNM-3**

Space group: *R*-3<br>$a = b = 43.38$ Å, and $c = 9.93$ Å<br>$\alpha = \beta = 90°$, and $\gamma = 120°$			
	X	Y	Z
Cu1	-0.6145	-0.30176	0.01248
C2	-0.40188	-0.7755	-0.01114
C3	-0.3476	-0.76752	-0.01192
C4	-0.38344	-0.79449	-0.01075
C5	-0.41767	-0.91101	0.04793
C6	-0.44086	-0.89883	0.00552
C7	-0.47671	-0.92542	-0.02306
C8	-0.48861	-0.96273	-0.01197
C9	-0.46447	-0.97376	0.02922
C10	-0.42921	-0.94759	0.05889
C11	-0.47588	-0.01068	0.04091
C12	-0.60413	-0.1659	0.00633

N13	-0.34528	-0.73524	-0.01266
N14	-0.26014	-0.638	-0.01232
N15	-0.4292	-0.8605	-0.00433
H16	-0.21321	-0.64367	-0.01002
H17	-0.6752	-0.22865	0.01143
H18	-0.39048	-0.8925	0.07571
H19	-0.49531	-0.91705	-0.0543
H20	-0.41032	-0.95544	0.09188
H21	-0.45701	-0.01869	0.07273
H22	-0.53442	-0.37455	0.00593

Table S4. The space group, cell parameters, total energy and total crystal stacking energy per layer of the four possible structures of **JNM-3**.

Structure	Space group	Formula	*c* (Å)	Total energy (kcal/mol)	Total crystal stacking energy per layer (kcal/mol)[a]
**JNM-3** adopts energetically favorable AA stacking (AA>ABC>AB)					
Monolayer		$C_{66}H_{42}N_{18}Cu_6$		-3048528.31	
AA-stacking	*P*6	$C_{66}H_{42}N_{18}Cu_6$	4.18	-3048574.80	46.49
AB-stacking	$P6_3$	$C_{132}H_{84}N_{36}Cu_{12}$	6.80	-6097128.10	35.73
ABC-stacking	*R*-3	$C_{198}H_{126}N_{54}Cu_{18}$	10.20	-9945704.70	39.91

[a] $\Delta \boldsymbol{E}_{AA/layer} = \boldsymbol{E}_{monolayer} - \boldsymbol{E}_{AA}$, $\Delta \boldsymbol{E}_{AB/layer} = (\boldsymbol{E}_{monolayer} \times 2 - \boldsymbol{E}_{AB})/2$, $\Delta \boldsymbol{E}_{ABC/layer} = (\boldsymbol{E}_{monolayer} \times 3 - \boldsymbol{E}_{ABC})/3$

Table S5. The space group, cell parameters, total energy and total crystal stacking energy per layer of the structures of protonated **JNM-3** with TFA.

Structure	Space group	Formula	*c* (Å)	Total energy (kcal/mol)	Total crystal stacking energy per layer (kcal/mol) [a]
**JNM-3** adopts energetically favorable AA-TFA stacking (AA-TFA>ABC-TFA)					
Monolayer		$C_{78}H_{48}N_{18}O_{12}F_{18}Cu_6$		-5030948.37	
AA-TFA stacking	*P*3	$C_{78}H_{48}N_{18}O_{12}F_{18}Cu_6$	4.31	-5031013.51	65.14
ABC-TFA stacking	*R*-3	$C_{234}H_{144}N_{54}O_{36}F_{54}Cu_{18}$	13.88	-15093027.68	60.85

[a] $\Delta \boldsymbol{E}_{AA\text{-}TFA/layer} = \boldsymbol{E}_{monolayer} - \boldsymbol{E}_{AA\text{-}TFA}$, $\Delta \boldsymbol{E}_{ABC\text{-}TFA/layer} = (\boldsymbol{E}_{monolayer} \times 3 - \boldsymbol{E}_{ABC\text{-}TFA})/3$

8. Gas adsorption isotherms and the pore size distribution

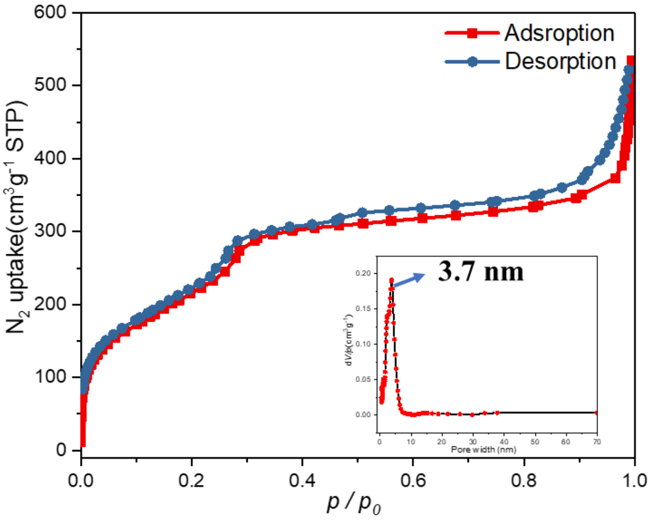

Figure S13. The N_2 adsorption and desorption isotherm profiles of **JNM-3-AA** at 77 K and the Brunauer−Emmett−Teller (BET) surface areas of **JNM-3-AA** are calculated to be 695.61 $m^2 \cdot g^{-1}$. Inset, the pore size distribution profiles of **JNM-3-AA** calculated by nonlocal DFT modeling based on N_2 adsorption data, showing a uniform pore size both of 3.7 nm.

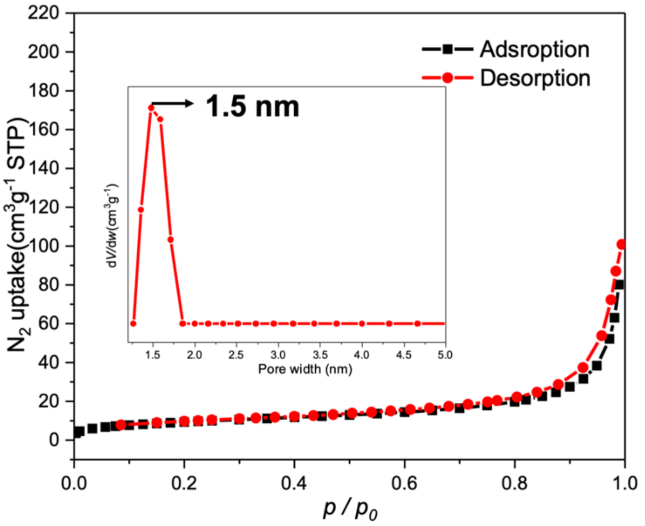

Figure S14. The N_2 adsorption and desorption isotherm profiles of **JNM-3-ABC** at 77 K and the Brunauer−Emmett−Teller (BET) surface areas of **JNM-3-ABC** are calculated to be 34.32 $m^2 \cdot g^{-1}$. Inset, the pore size distribution profiles of **JNM-3-ABC** calculated by nonlocal DFT modeling based on N_2 adsorption data, showing a uniform pore size both of ~1.5 nm.

9. Transmission electron microscopy (TEM)

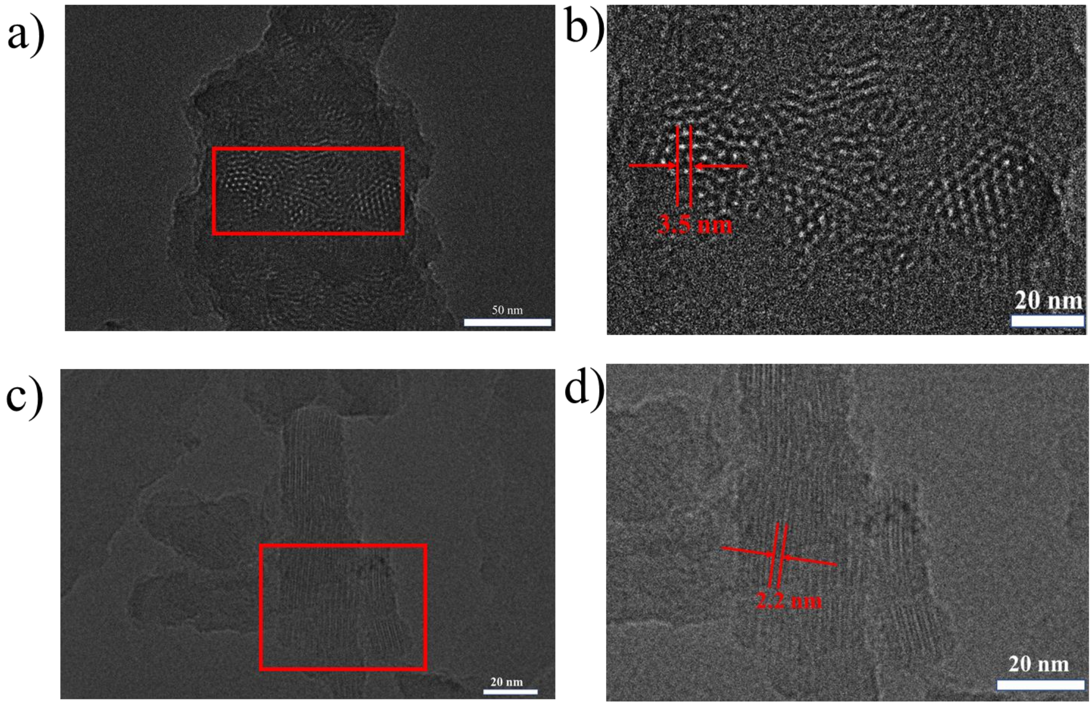

Figure S15. (a, b) TEM images of **JNM-3-AA**; and (c, d) TEM images of **JNM-3-ABC**.

10. Thermogravimetric analysis (TGA)

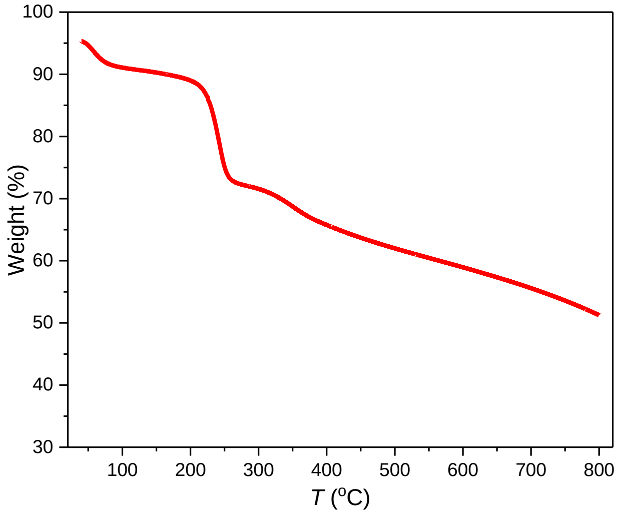

Figure S16. TGA curve of **JNM-3-AA** under N_2 atmosphere.

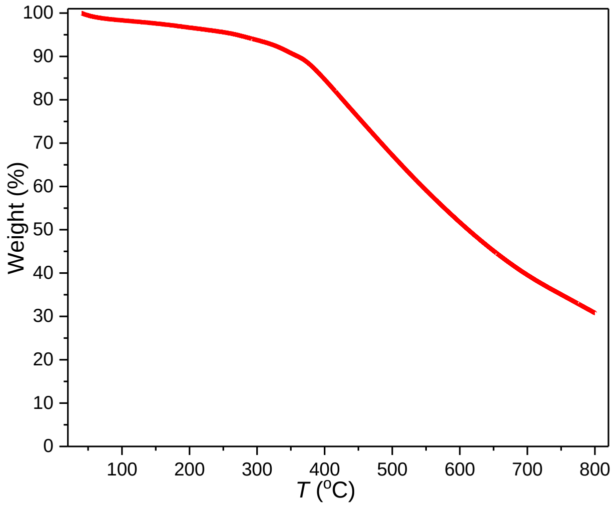

Figure S17. TGA curve of **JNM-3-ABC** under N_2 atmosphere.

11. Various-Temperature PXRD

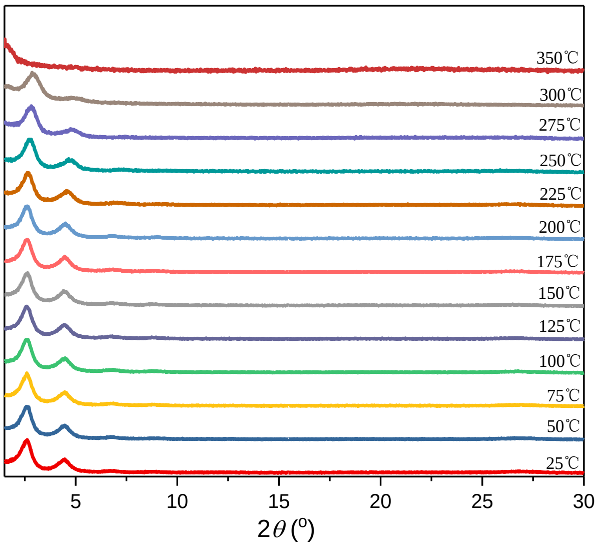

Figure S18. *In-situ* variable-temperature PXRD patterns of **JNM-3-AA** under a N_2 atmosphere.

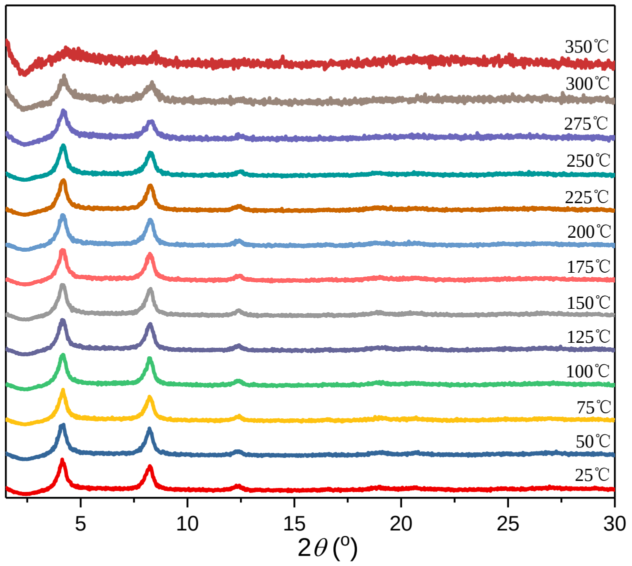

Figure S19. *In-situ* variable-temperature PXRD patterns of **JNM-3-ABC** under a N_2 atmosphere.

12. X-ray photoelectron spectroscopy (XPS)

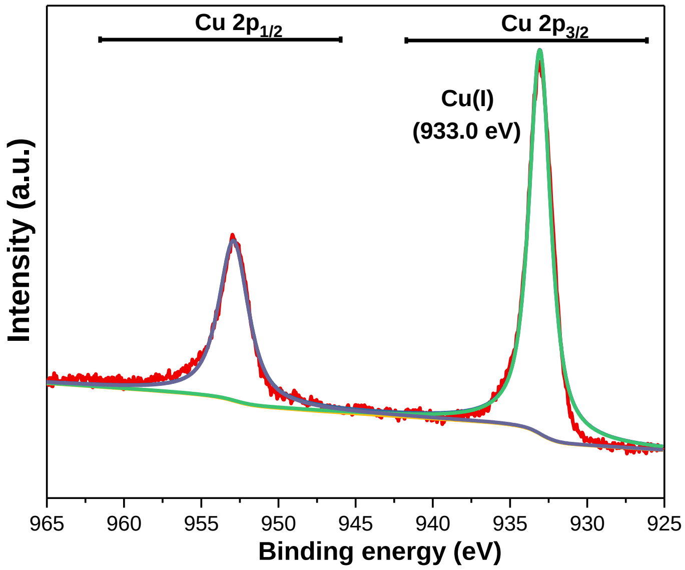

Figure S20. XPS spectrum of **JNM-3-AA** exposed to air for over one month.

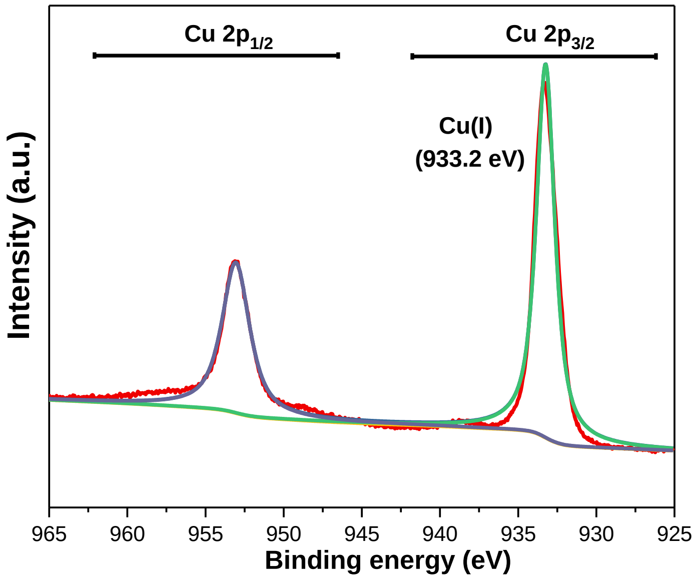

Figure S21. XPS spectrum of **JNM-3-ABC** exposed to air for over one month.

13. Stability in various solvents

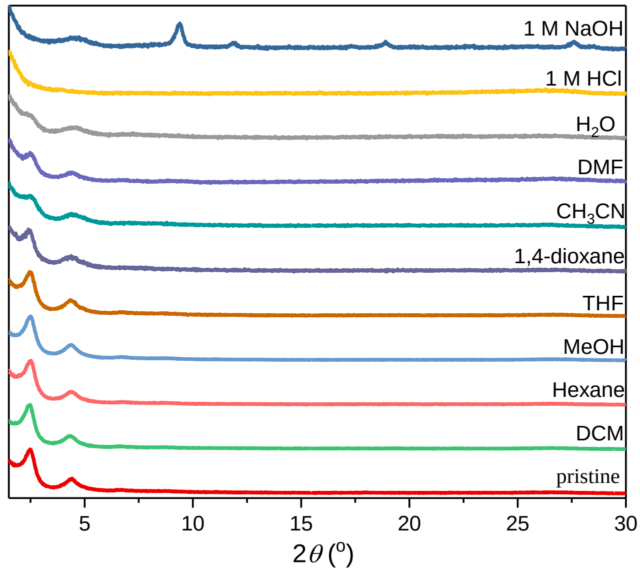

Figure S22. PXRD patterns for samples of **JNM-3-AA** after treatment with different solvents for 3 d.

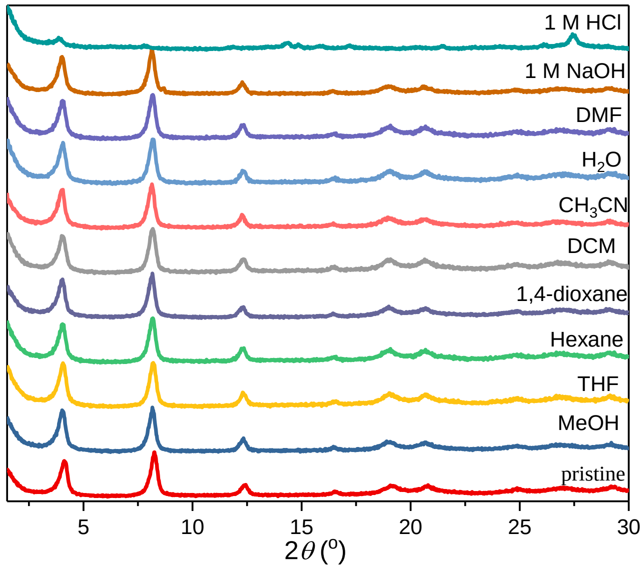

Figure S23. PXRD patterns for samples of **JNM-3-ABC** after treatment with different solvents for 3 d.

14. Structure transformation

Experimental procedures: **JNM-3-ABC** (20 mg), mesitylene (0.5 mL) and 1,4-dioxane (0.5 mL) with varied TFA concentration (0, 0.1, 0.2, 0.3, 0.4, and 0.5 μM) was added into a glass ampoule. The resulted mixture was stirred at room temperature for 30 mins., and then the solid was isolated by filtration, washed and solvent exchanged with EtOH, DMF and acetone for several times. Resulted powder was dried under vacuum at room temperature for 4 h, and then was carried on the PXRD analysis.

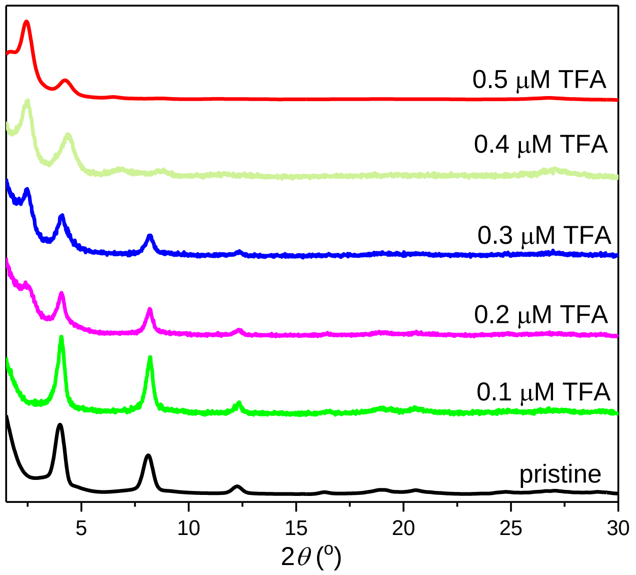

Figure S24. Monitoring the interlayer stacking transformation by adding varied concentration of TFA (black, pristine; green to red, 0.1 to 0.5 μM TFA).

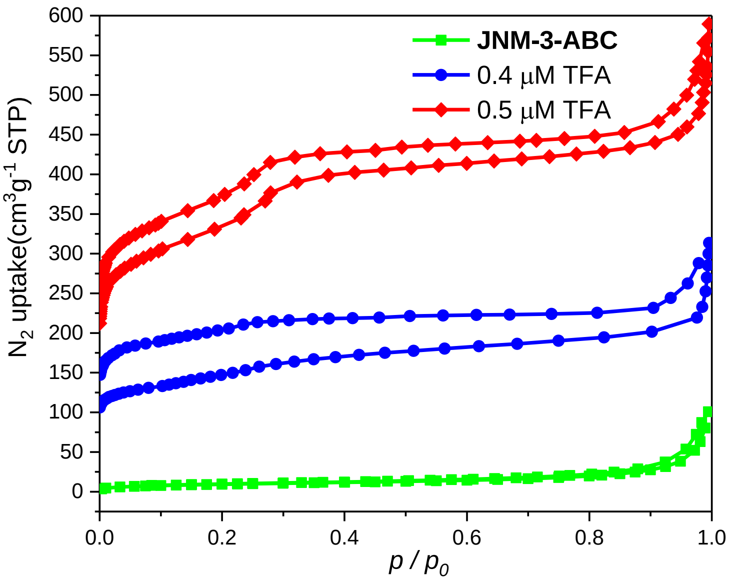

Figure S25. The N_2 adsorption and desorption isotherm profiles of **JNM-3-ABC** (green), 0.4 μM TFA (blue) and 0.5 μM TFA (red) at 77K. Their corresponding Brunauer−Emmett−Teller (BET) surface areas are calculated to be 34.22, 199.87 and 452.22 $m^2 \cdot g^{-1}$, respectively.

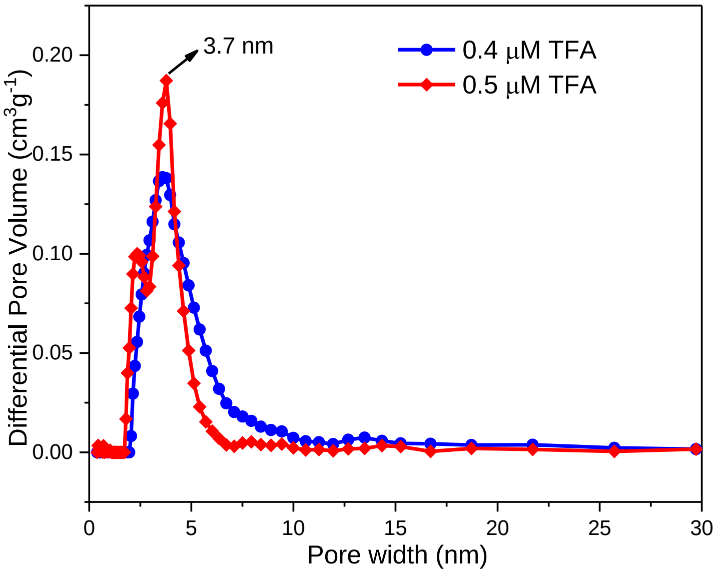

Figure S26. The pore size distribution profiles of transferred samples from JNM-3-ABC after adding 0.4 μM TFA (blue) and 0.5 μM TFA (red) and both of them show a narrow pore size distribution of ~3.7 nm.

Neutralization of JNM-3-AA with base: JNM-3-AA (20 mg) were immersed in a methanol solution of CH_3ONa (0.5 M, 10 mL) for 3 days, and the deep red powders turned to yellow. The resulted yellow solids were washed with ethanol, DMF, and acetone for several times and dried at 80 ℃ for 10 hours. The PXRD of resulted powders are similar to that of **JNM-3-AA** indicating no structure transformation occurred during the neutralization.

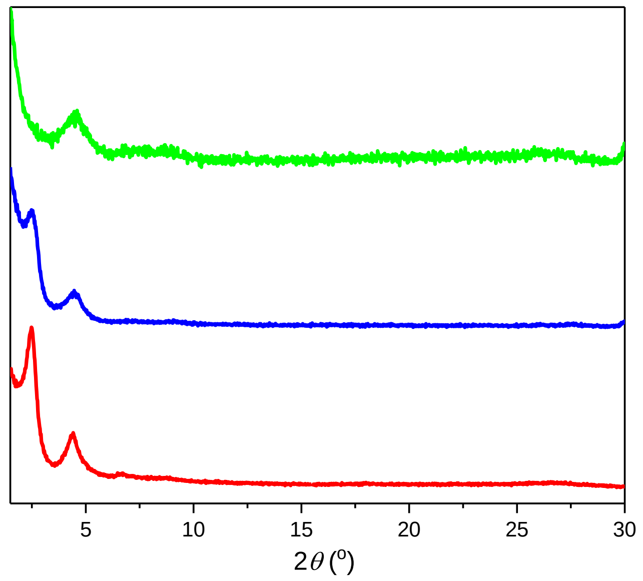

Figure S27. PXRD patterns of **JNM-3-AA** (before, red; 0.5 M CH_3ONa, blue; and 1 M CH_3ONa, green).

15. Dye adsorption experiments

Initial concentrations of chrome azurol S (CA) were fixed to be 100 μM. Typically 10 mg of **JNM-3-ABC** without TFA (6M, 0.2 mL) or with TFA (6M, 0.2 mL) was added into aqueous solution of CA (3 mL), then the mixture was stirred at rt. At appropriate time interval, the mixture was filtered in syringes equipped with Whatman 0.45 μm membrane filters. The concentration of CA in the filtrate was detected using an ultraviolet-visible spectrometer at a wavelength of maximum absorbance.

The removal efficiency of dye was calculated as following equation (1):

$$\text{Removal efficiency (\%)} = (C_0 - C_t) / C_0 \times 100 \qquad (1)$$

Where C_0 and C_t are the concentration of dyes at initial condition and in the filtrate, respectively.

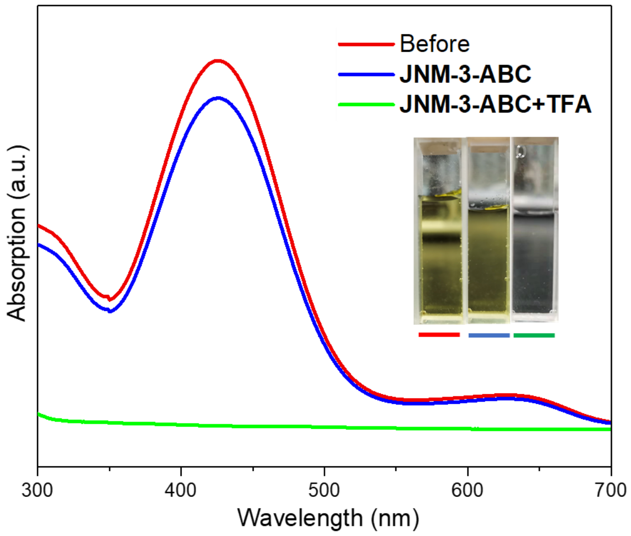

Figure S28. Uv-vis absorption spectra of an aqueous solution of chrome azurol S (CA), indicating slight decrease or vanish of absorption intensity after treatment with **JNM-3-ABC** or **JNM-3-ABC** and TFA, respectively. (orange and blue line represents before and after treatment with **JNM-3-ABC** at 3 mins; red line represents after addition of TFA into the mixture of **JNM-3-ABC** and CA for 3 mins; inset, the photographs of CA, and filtrate after treatment of only **JNM-3-ABC** or **JNM-3-ABC** and TFA, showing yellow, yellow, and colorless).

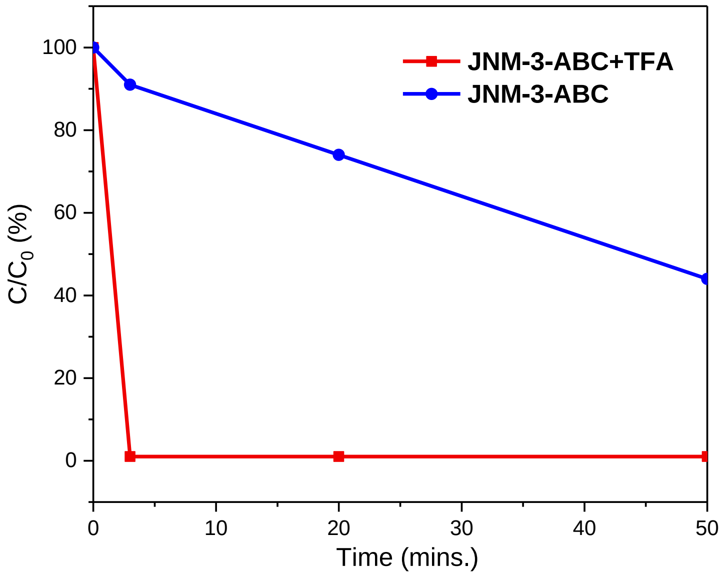

Figure S29. Change in concentration of CA over time after treatment with **JNM-3-ABC** or **JNM-3-ABC** and TFA, determined by change in absorbance relative to initial absorbance (C/C_0).

16. Catalysis for Click reaction

16-1. Theoritical density of metal open sites in JNM-3-AA and JNM-3-ABC.

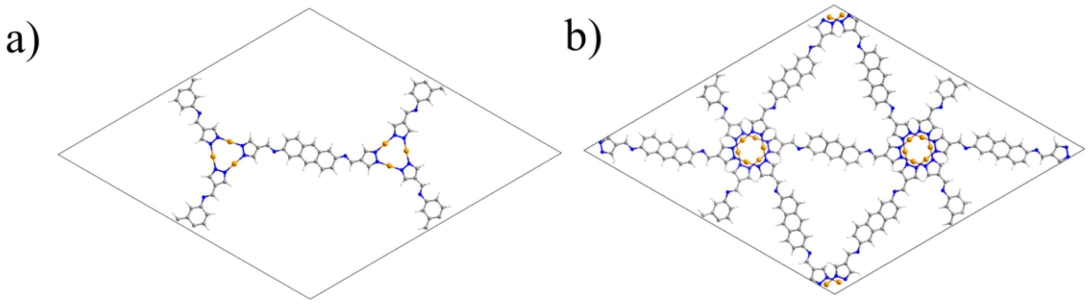

Figure S30. Illustrations for the single cell of (a) **JNM-3-AA** and (b) **JNM-3-ABC**.

As shown in Figure S30, in unit cell, there are 6 and 18 copper(I) ions in **JNM-AA** and **JNM-3-ABC**, respectively. Thus, the theoretical ratio of copper(I) density between **JNM-3-ABC** and **JNM-3-AA** can be calculated as following equation:

$$\textbf{JNM-3-ABC}: \textbf{JNM-3-AA} = 3/(V_{ABC}/V_{AA}) = 7{:}6 \qquad (2)$$

16-2. Experimental details for Click reaction catalyzed by JNM-3

General Procedures: Before the catalytic experiment, the catalysts were dried in vacuum at 100 °C for 8 h. 4 mol% of the dried catalysts (base on Cu-CTU) and 3 mL of solvent were added into a 10 mL Purex tube. Then alkyne (0.5 mmol), methyl 2-azidoacetate (0.6 mmol, 69.2 mg) were added into the tube, respectively. The mixture were stirred under N_2 atmosphere for 12 h. After that, 50 μL of the reaction liquid was taken and diluted with CH_3CN to 1 mL. The solvent was analyzed by GC-MS. The reaction conversion was calculated based on the alkyne substrate. After the reaction was completed, the reaction mixture was quenched with water. The aqueous layer was extracted with ethyl acetate (3 × 20 mL) and the combined organic layers were washed with water, dried with anhydrous $MgSO_4$, filtered, and concentrated under reduced pressure. The resulting residue was purified by column chromatography over silica gel using an increasing EA/hexane gradient to afford desired pure products.

Table S6. Optimization for Click reaction

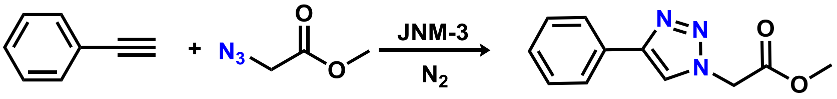

Entry	Catalyst	Temperature/℃	Solvent	Conversion[a](%)
1	**JNM-3-AA**	rt	DCM	10
2	**JNM-3-ABC**	rt	DCM	81
3	**JNM-3-AA**	60	*n*-BuOH	90
4	**JNM-3-ABC**	40	DCM	93
5	none	rt	DCM	0
6	CTU **1**	rt	DCM	99

[a]Reaction conditions: 0.5 mmol phenylacetylene, 1.2 equiv of methyl 2-azidoacetate, 4 mol% of the catalyst (based on Cu-CTU), solvent (3 mL), 12 h, N_2 atm. The reported conversions in this table are based on GC-MS analysis.

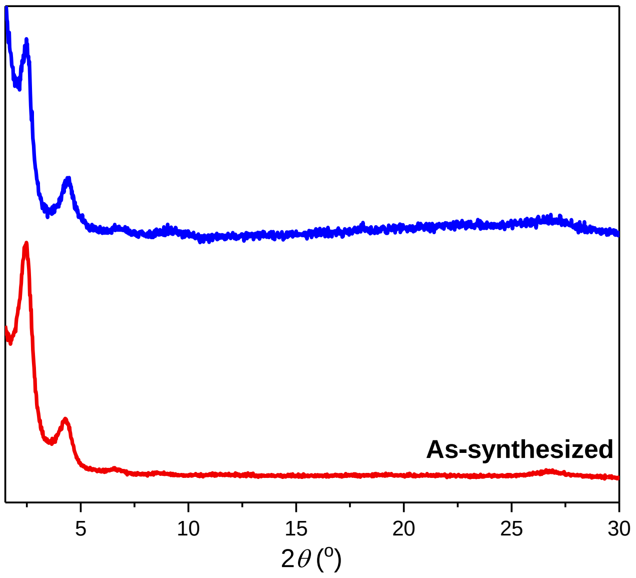

Figure S31. PXRD patterns of As-synthesized **JNM-3-AA** (red) and **JNM-3-AA** after catalytic reaction (blue).

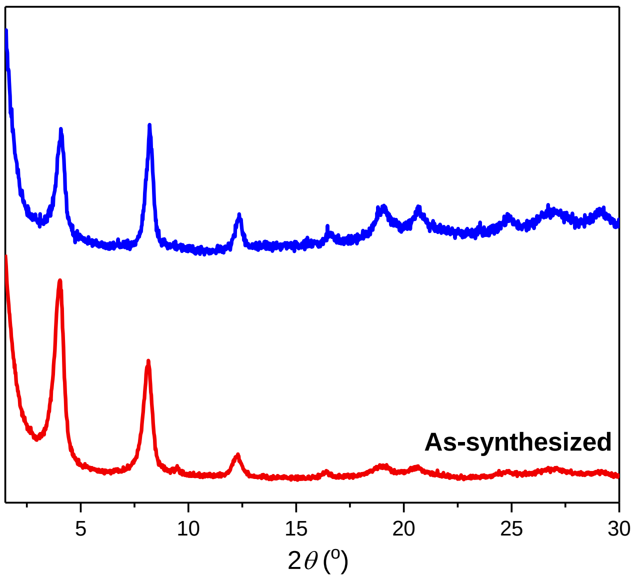

Figure S32. PXRD patterns of As-synthesized **JNM-3-ABC** (red) and **JNM-3-ABC** after catalytic reaction (blue).

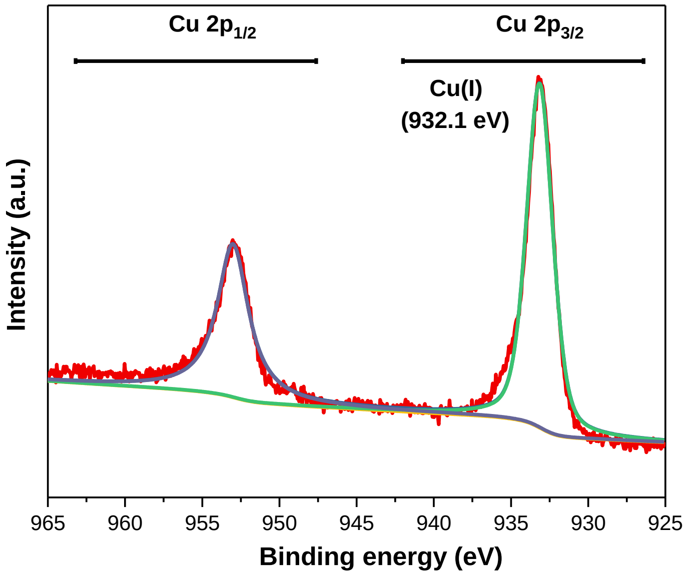

Figure S33. XPS spectrum of **JNM-3-AA** after catalytic reaction.

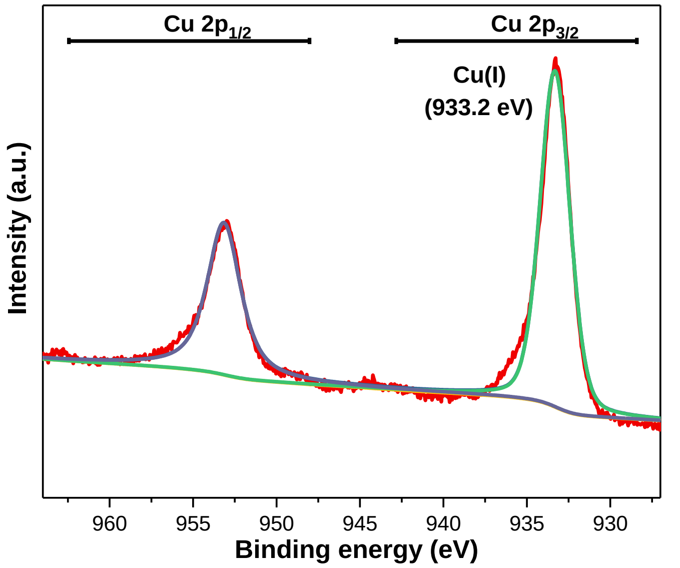

Figure S34. XPS spectrum of **JNM-3-ABC** after catalytic reaction.

Table S7. Scope of JNM-3 Catalyst for Click Reaction

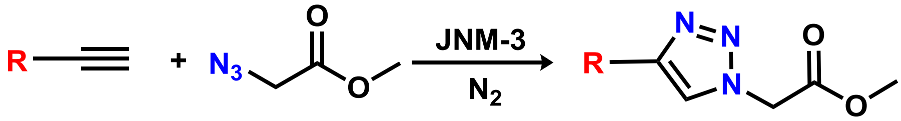

Entry	Product	**JNM-3-ABC** (Yields) [b]	**JNM-3-AA** (Yields) [a]	CTU **1** (Yields) [b]	**JNM-3-AA** (Yields) [b, c]
1	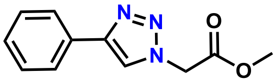	79	8	98	88
2	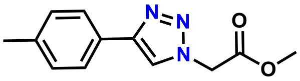	70	11	99	90
3	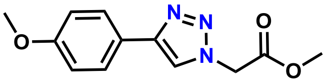	66	10	97	86
4	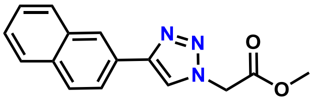	36	13	97	93
5	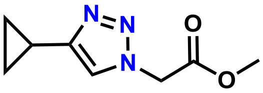	86	9	40	90
6	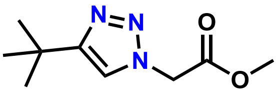	65	4	51	83
7	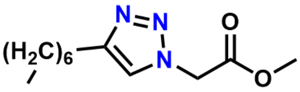	10	2	<1	61

[a,b]Reaction conditions: 0.5 mmol alkyne, 1.2 equiv of methyl 2-azidoacetate, 4 mol% of the catalyst (base on Cu-CTU), DCM (3 mL), rt, 12 h, N_2 atm. The reported yields here are based on [a]GC-MS analysis, and [b]isolated yields, respectively. [c]Reaction conditions: instead of DCM at rt, here the solvent has been changed as *n*-BuOH (3 mL) at 60℃.

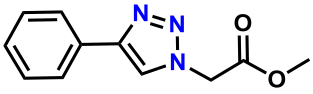 **Methyl 2-(4-phenyl-1H-1,2,3-triazol-1-yl) acetate.**

^{1}H NMR (400 MHz, 298K, $CDCl_3$) δ 7.95 (s, 1H), 7.81 (d, J = 7.5 Hz, 2H), 7.45–7.30 (m, 3H), 5.19 (s, 2H), 3.75 (s, 3H) ppm. ^{13}C NMR (400 MHz, 298K, $CDCl_3$) δ 166.88, 130.30, 128.86, 128.30, 125.75, 53.02, 50.77 ppm.

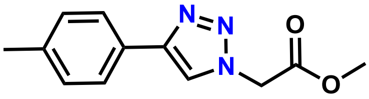 **Methyl 2-(4-(p-tolyl)-1H-1,2,3-triazol-1-yl) acetate.**

^{1}H NMR (400 MHz, 298K, $CDCl_3$) δ 7.89 (s, 1H), 7.75 (d, J = 7.9 Hz, 2H), 7.25 (d, J = 7.9 Hz, 2H), 5.22 (s, 2H), 3.83 (s, 3H), 2.40 (s, 3H) ppm. ^{13}C NMR (400 MHz, 298K, $CDCl_3$) δ 166.77, 148.38, 138.17, 129.53, 127.51, 125.73, 53.05, 50.79, 21.29 ppm.

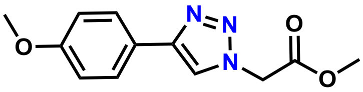 **Methyl 2-(4-(4-methoxyphenyl)-1H-1,2,3-triazol-1-yl) acetate.**

^{1}H NMR (400 MHz, 298K, $CDCl_3$) δ 7.84 (s, 1H), 7.77 (d, J = 8.0 Hz, 2H), 6.97 (d, J = 8.0 Hz, 2H), 5.21 (s, 2H), 3.85 (s, 3H), 3.82 (s, 3H) ppm. ^{13}C NMR (400 MHz, 298K, $CDCl_3$) δ 166.84, 159.68, 148.13, 127.12, 123.01, 120.25, 114.25, 55.34, 53.08, 50.80 ppm.

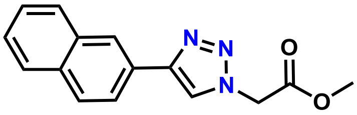 **Methyl 2-(4-(naphthalen-2-yl)-1H-1,2,3-triazol-1-yl) acetate.**

^{1}H NMR (400 MHz, 298K, $CDCl_3$) δ 8.40 (s, 1H), 8.07 (s, 1H), 7.93–7.96 (m, 3H), 7.87 (d, J = 8.7 Hz, 1H), 7.55–7.48 (m, 2H), 5.29 (s, 2H), 3.87 (s, 3H) ppm. ^{13}C NMR (400 MHz, 298K, $CDCl_3$) δ 166.73, 133.52, 133.23, 128.64, 128.25, 127.80, 127.64, 126.51, 126.26, 124.62, 123.85, 53.18, 50.94 ppm.

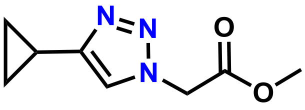 **Methyl 2-(4-cyclopropyl-1H-1,2,3-triazol-1-yl) acetate.**

^{1}H NMR (400 MHz, 298K, $CDCl_3$) δ 7.39 (s, 1H), 5.08 (s, 2H), 3.74 (s, 3H), 0.92–0.87 (m, 2H), 0.83–0.76 (m, 2H) ppm. ^{13}C NMR (400 MHz, 298K, $CDCl_3$) δ 166.95, 52.91, 50.69, 18.91, 13.86, 7.67, 6.69 ppm.

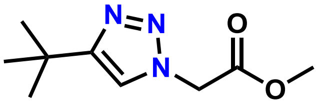

Methyl 2-(4-(tert-butyl)-1H-1,2,3-triazol-1-yl) acetate.

^{1}H NMR (400 MHz, 298K, $CDCl_3$) δ 7.40 (s, 1H), 5.09 (s, 2H), 3.72 (s, 3H), 1.30 (s, 9H) ppm. ^{13}C NMR (400 MHz, 298K, $CDCl_3$) δ 167.08, 52.81, 52.50, 50.49, 50.11, 30.70, 30.20 ppm.

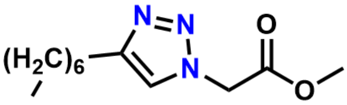

Methyl 2-(4-heptyl-1H-1,2,3-triazol-1-yl) acetate.

^{1}H NMR (400 MHz, 298K, $CDCl_3$) δ 7.41 (s, 1H), 5.09 (s, 2H), 3.72 (s, 3H), 2.66 (t, *J* = 7.6 Hz, 2H), 1.69–1.57 (m, 2H), 1.32–1.18 (m, 8H), 0.81 (t, *J* = 6.7 Hz, 3H) ppm. ^{13}C NMR (400 MHz, 298K, $CDCl_3$) δ 167.01, 52.48, 50.54, 50.12, 31.68, 29.27, 29.12, 28.96, 25.57, 22.56, 13.99 ppm.

REFERENCES

(1) X. Chen, M. Addicoat, S. Irle, A. Nagai and D. Jiang, *J. Am. Chem. Soc.*, 2013, **135**, 546-549.

(2) P. Albacete, J. I. Martinez, X. Li, A. Lopez-Moreno, S. A. Mena-Hernando, A. E. Platero-Prats, C. Montoro, K. P. Loh, E. M. Perez and F. Zamora, *J. Am. Chem. Soc.*, 2018, **140**, 12922-12929.
